# Mathematical model of oxygen, nutrient, and drug transport in tuberculosis granulomas

**DOI:** 10.1371/journal.pcbi.1011847

**Published:** 2024-02-09

**Authors:** Meenal Datta, McCarthy Kennedy, Saeed Siri, Laura E. Via, James W. Baish, Lei Xu, Véronique Dartois, Clifton E. Barry, Rakesh K. Jain

**Affiliations:** 1 Edwin L. Steele Laboratories, Department of Radiation Oncology, Massachusetts General Hospital and Harvard Medical School, Boston, Massachusetts, United States of America; 2 Department of Aerospace and Mechanical Engineering, University of Notre Dame, Notre Dame, Indiana, United States of America; 3 Department of Chemical and Biomolecular Engineering, University of Notre Dame, Notre Dame, Indiana, United States of America; 4 Tuberculosis Research Section, Laboratory of Clinical Immunology and Microbiology, National Institute of Allergy and Infectious Disease (NIAID), National Institutes of Health, Bethesda, Maryland, United States of America; 5 Department of Biomedical Engineering, Bucknell University, Lewisburg, Pennsylvania, United States of America; 6 Center for Discovery and Innovation, Hackensack Meridian School of Medicine, Hackensack Meridian Health, Nutley, New Jersey, United States of America; Utrecht University, NETHERLANDS

## Abstract

Physiological abnormalities in pulmonary granulomas–pathological hallmarks of tuberculosis (TB)–compromise the transport of oxygen, nutrients, and drugs. In prior studies, we demonstrated mathematically and experimentally that hypoxia and necrosis emerge in the granuloma microenvironment (GME) as a direct result of limited oxygen availability. Building on our initial model of avascular oxygen diffusion, here we explore additional aspects of oxygen transport, including the roles of granuloma vasculature, transcapillary transport, plasma dilution, and interstitial convection, followed by cellular metabolism. Approximate analytical solutions are provided for oxygen and glucose concentration, interstitial fluid velocity, interstitial fluid pressure, and the thickness of the convective zone. These predictions are in agreement with prior experimental results from rabbit TB granulomas and from rat carcinoma models, which share similar transport limitations. Additional drug delivery predictions for anti-TB-agents (rifampicin and clofazimine) strikingly match recent spatially-resolved experimental results from a mouse model of TB. Finally, an approach to improve molecular transport in granulomas by modulating interstitial hydraulic conductivity is tested *in silico*.

## Author summary

Treatment failure in infectious diseases such as tuberculosis is often the result of inadequate drug delivery to the site of infection. In the case of tuberculosis, that site is most commonly a pulmonary granuloma–an abnormal mass of immune cells that forms in the lung in response to the body’s attempt to confine and constrain the infection-causing bacteria. Within the granuloma interior, blood vessels are non-functional or absent, and the tissue is dense and fibrous. These physiological abnormalities hinder not only drug delivery, but also oxygen and nutrient availability to the immune cells fighting off the infection. Here, we mathematically examine the basis and scope of the poor delivery of oxygen, glucose, and tuberculosis-fighting drugs in granulomas. Our simulations agree with experimental results from animal models of this disease and reveal potential strategies to overcome physiological barriers to drug delivery in granulomas.

## Introduction

Tuberculosis (TB) afflicts roughly one-third of the global population and causes approximately 1.5 million deaths annually [1]. Its treatment often requires a lengthy drug therapy, in part because the dormant *Mycobacterium tuberculosis* bacilli hide within the core of hardened cellular masses in lungs called granulomas that offer a significant barrier to the transport of nutrients and therapeutic drugs. It is, thus, important to understand the flux of small molecules within granulomas, so that strategies might be devised to overcome transport limitations and hence improve treatment outcomes.

In order to reach the granuloma-contained bacilli, drugs must travel via the circulatory system to the lesion site, exit the granuloma vasculature (i.e., transvascular transport), and traverse the granuloma interior (i.e., interstitial transport) before encountering its target (i.e., cellular metabolism) [2]. A related area of study wherein the path of circulating agents to and through abnormal masses has been thoroughly explored is the field of tumor transport [3,4]. Indeed, TB granulomas and cancerous solid tumors are morphologically similar, resulting in similar transport limitations that have implications for therapeutic delivery and efficacy. As we have previously reported, a shared characteristic of these two cellular structures is an abnormal vasculature that is non-uniformly distributed within the tissue, resulting in avascular, diffusion-dominated regions, and thus, poor oxygen and drug delivery [5].

Within tumors, transvascular transport is compromised by the poorly formed and leaky tumor vasculature [6]. This negatively impacts interstitial transport, exacerbated further by a lack of functional lymphatics, which in normal physiological settings serve a crucial role in homeostatic fluid balance within tissues [7]. Impaired drainage by non-functional lymph vessels combined with abnormal, hyper-permeable blood vessels, results in an overall reduced pressure difference between the blood vessel and the interstitium [7], which is the driving force for transcapillary plasma exchange. This increases the interstitial fluid pressure (IFP), thereby reducing the effectiveness of the convective delivery of oxygen, nutrients, and drugs from the blood vessels within the core of the tumors, and hence providing a considerable barrier to effective delivery of anti-tumor agents. Although an absence of lymph vessels has not been confirmed within TB granuloma masses, we posit here that a similar IFP rise occurs within TB granulomas, as we have previously shown that granuloma blood vessels are structurally and functionally abnormal in the same manner as tumor blood vessels–and can even be “normalized” using the same targeted pharmacological approaches as for cancer [5].

The rise in IFP toward the core causes a radially outward convective flow of interstitial fluid velocity (IFV) oozing out at the periphery, thwarting the inward diffusion of drugs and nutrients [8,9]. Interstitial diffusion, particularly of macromolecules, is further impeded by a dense cellular matrix. This has motivated many studies of tumor transport so that impediments to anti-cancer therapies can be understood and strategies developed to overcome them [10–14]. Although IFP and IFV have not yet been directly measured within granulomas as they have been in tumors, we posit that a similar phenomenon occurs in TB, as we have found that other aspects including vasculature and hypoxia have been found to be analogous between the two lesion types in our prior experimental work [5].

The observable physiological abnormalities motivate a better understanding of the disease etiology and the underlying physicochemical properties that result in granuloma transport limitations. We first addressed this via a mathematical analysis of interstitial *diffusion* to quantitatively describe the emergence of hypoxic and necrotic granuloma regions as a direct result of limited oxygen availability [15]. We now build upon these findings to include both *convection and diffusion* in the variably perfused regions in the granuloma microenvironment (GME). Because of the morphological similarities between tumors and granulomas, the substantial tumor transport modeling and experimental literature was leveraged to guide this approach; thus, results are shown for both abnormal masses.

## Materials and methods

**Fig 1:**
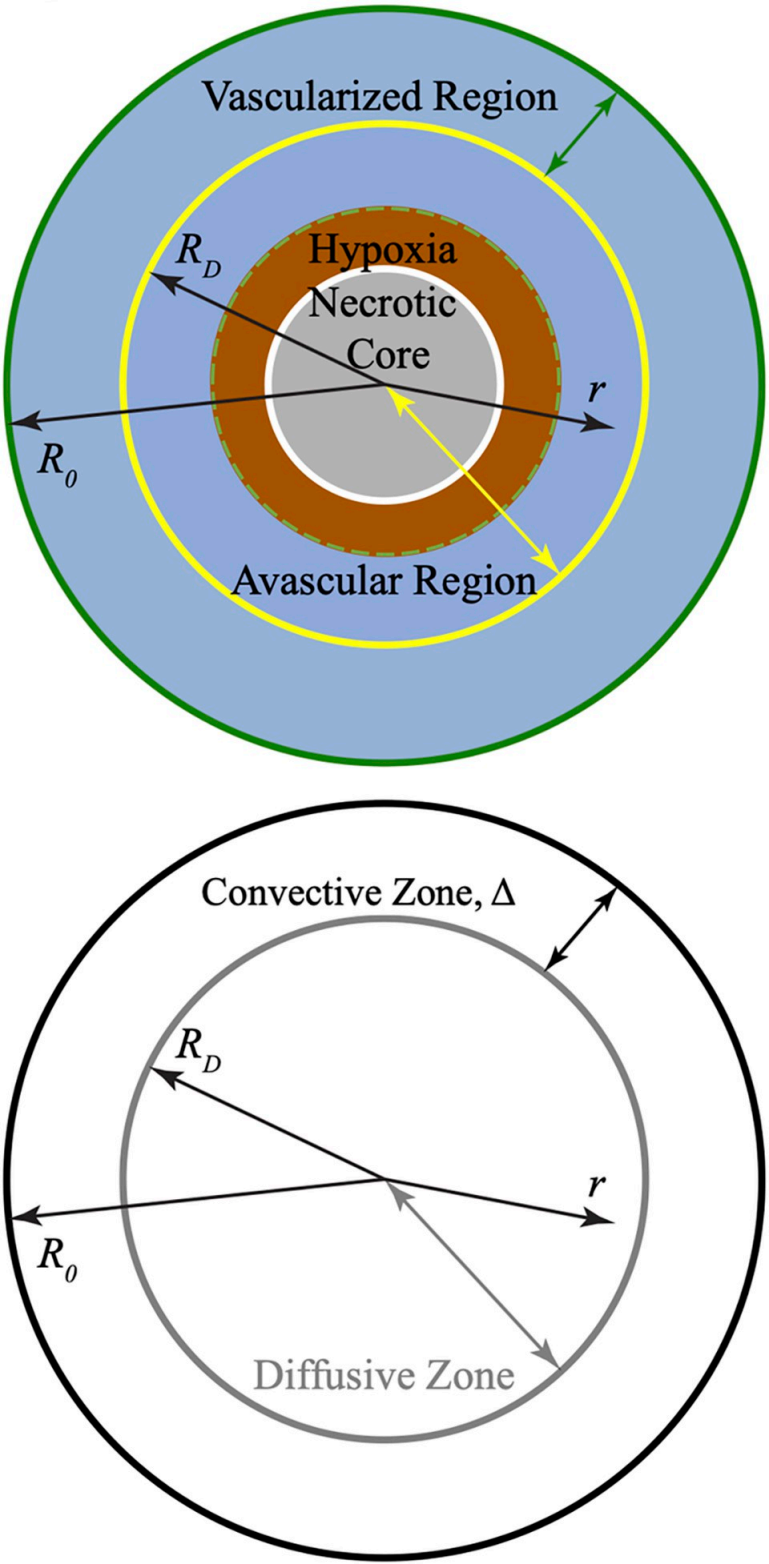
Physiological basis for compartmentalized transport models in TB granulomas. Schematic depicting regions and consequences of compromised oxygen transport in idealized spherical granulomas, including 1) a vascularized region where convection dominates and plasma filtration from blood vessels occurs, and 2) an inner region lacking blood vessels where diffusion dominates, and hypoxia and necrosis result. (Adapted from [15]).

**Fig 2:**
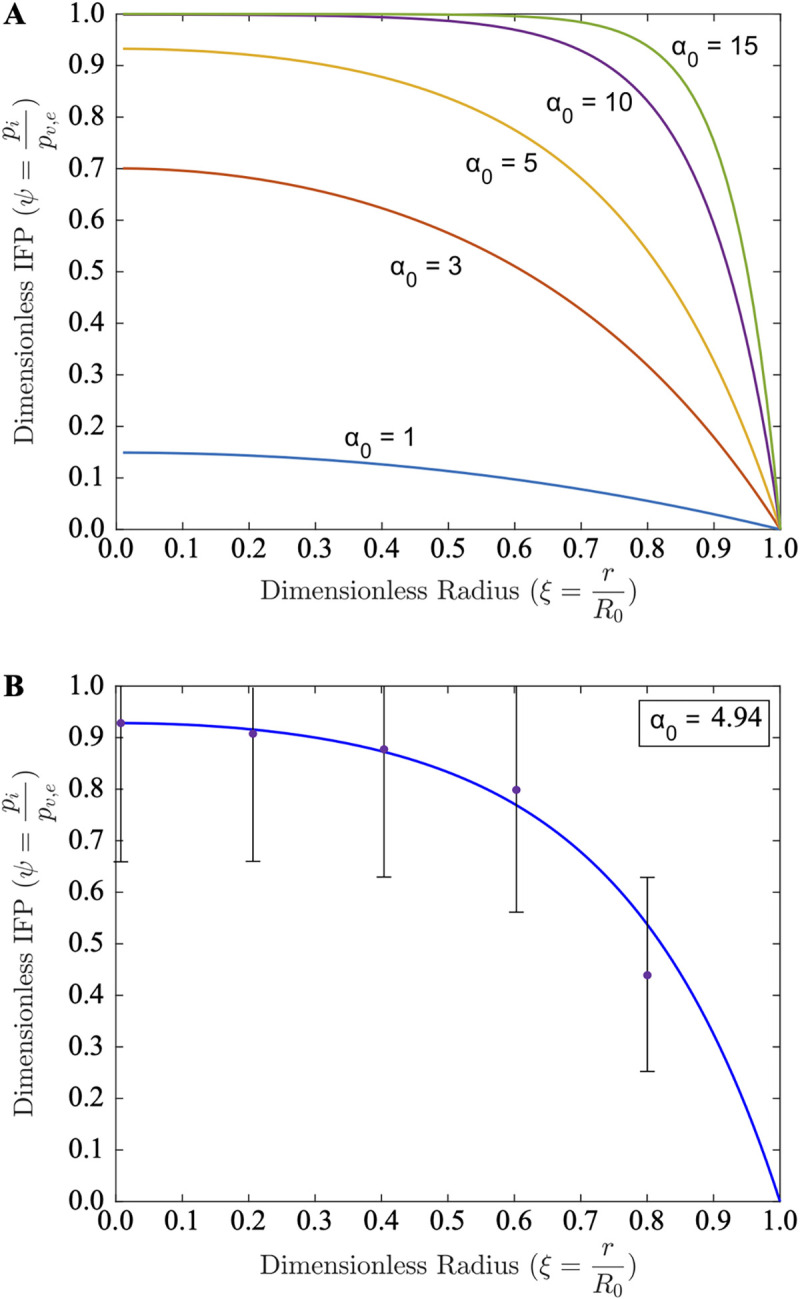
Granuloma IFP estimates and comparisons to tumor data. (**A**) Predicted dimensionless IFP profiles within granulomas from the uniform perfusion case for varying values of dimensionless granuloma size, *α*_0_ = 1–15. (**B**) Fitting the theoretical IFP estimates (Eq III with a fitted modulus $\alpha_{0}^{2}=24.4$ [9], see S1 Text) to experimentally measured tumor IFP data (from human neuroblastoma tumor models grown in immunosuppressed rats, ~2 cm in diameter [9]) demonstrates the applicability of the uniform perfusion model to physiological IFP levels with a single fitted parameter.

**Fig 3:**
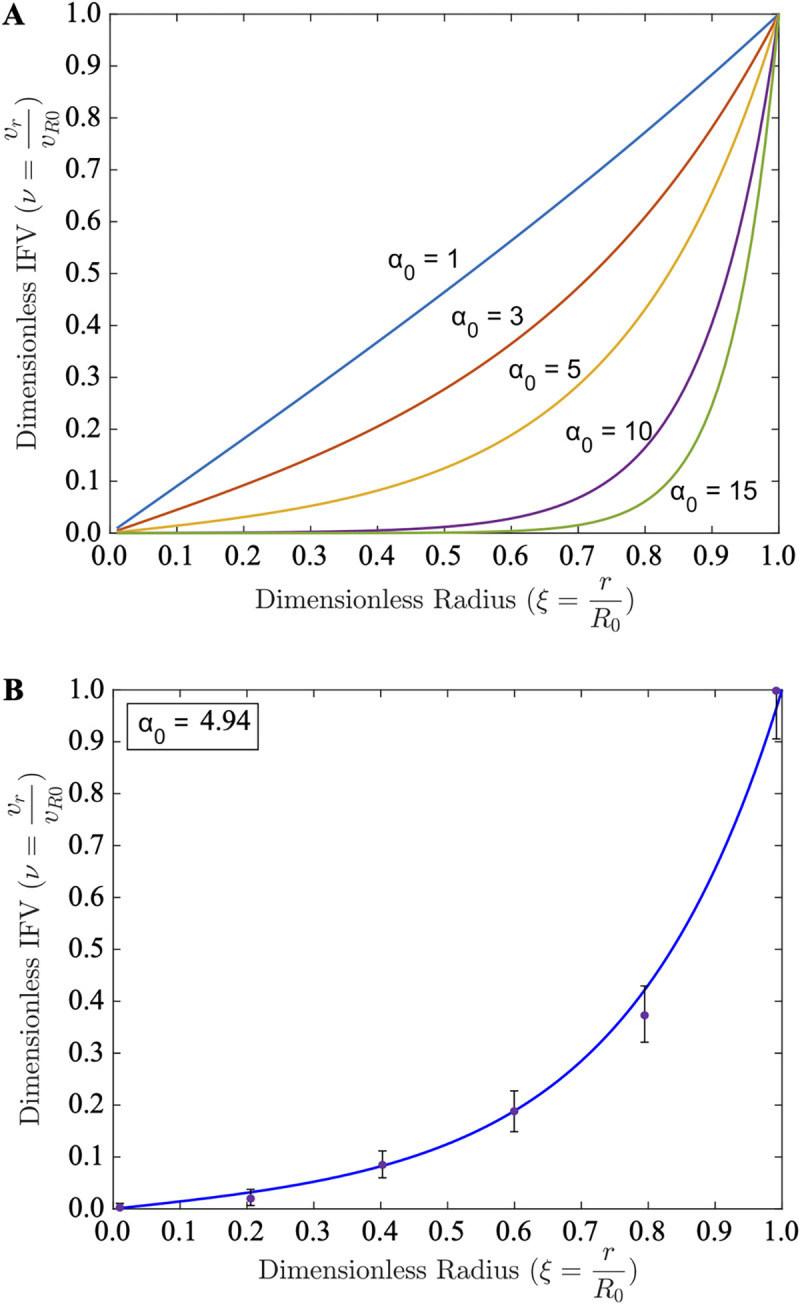
Granuloma IFV estimates and comparisons to tumor data. (**A**) Predicted dimensionless IFV profiles within granulomas from the uniform perfusion case for varying values of dimensionless granuloma size, *α*_0_ = 1–15. (**B**) Fitting the theoretical IFV estimates (Eq IV with a fitted modulus $\alpha_{0}^{2}=24.4$ [9], see S1 Text) to experimentally measured tumor IFV data [9] demonstrates the applicability of the uniform perfusion model to physiological IFV levels with a single fitted parameter.

**Fig 4:**
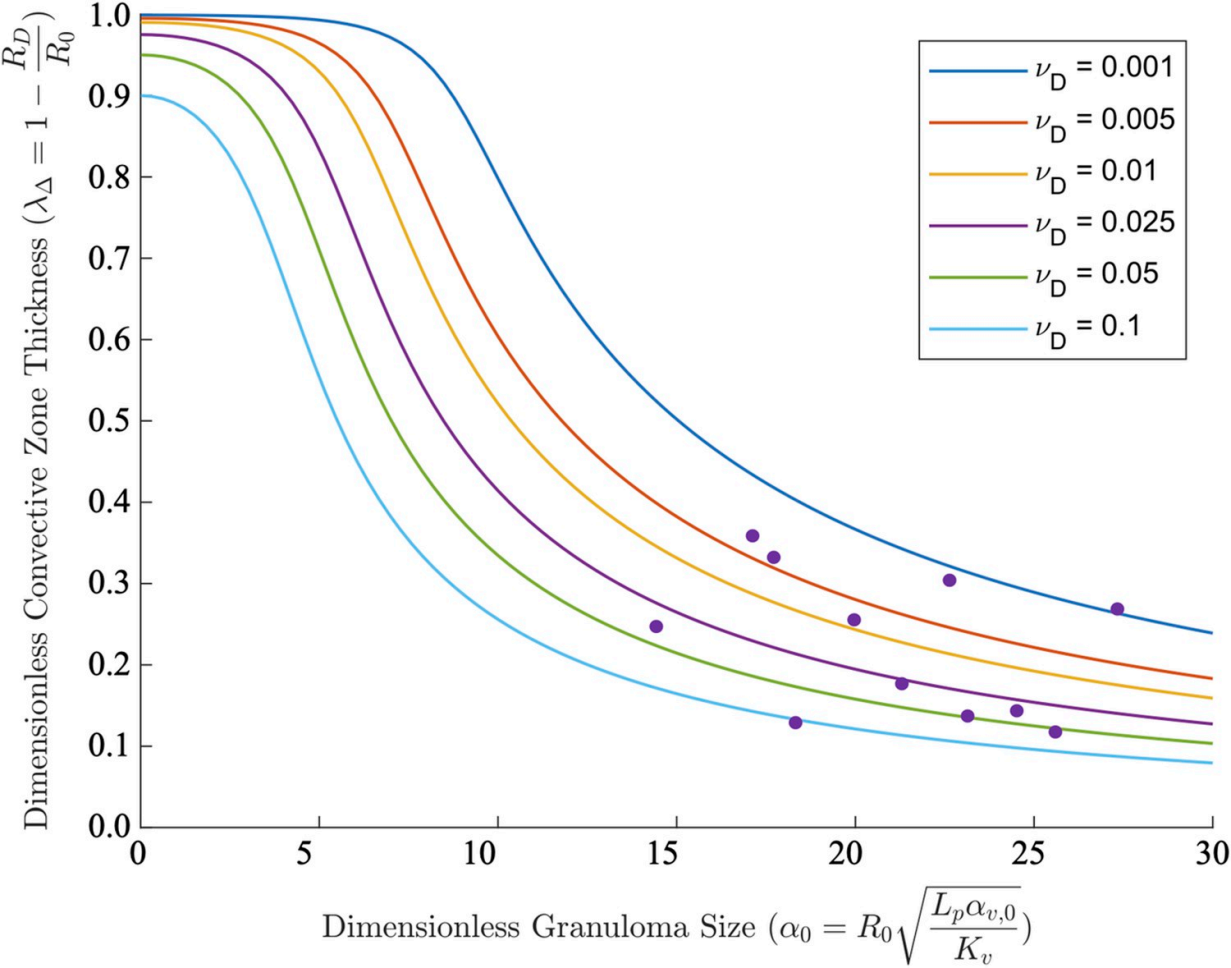
Granuloma convective zone thickness estimates and comparisons to experimental granuloma data. Dimensionless convective zone thickness, *λ*_Δ_ (lines), as a function of dimensionless granuloma size (0 < *α*_0_ < 30) for varying values of dimensionless limiting perfusion velocity, $v_{D}/v_{R_{0}}$ (as defined in the S1 Text), in comparison to experimental data from rabbit TB granulomas [15] (gray dots).

**Fig 5:**
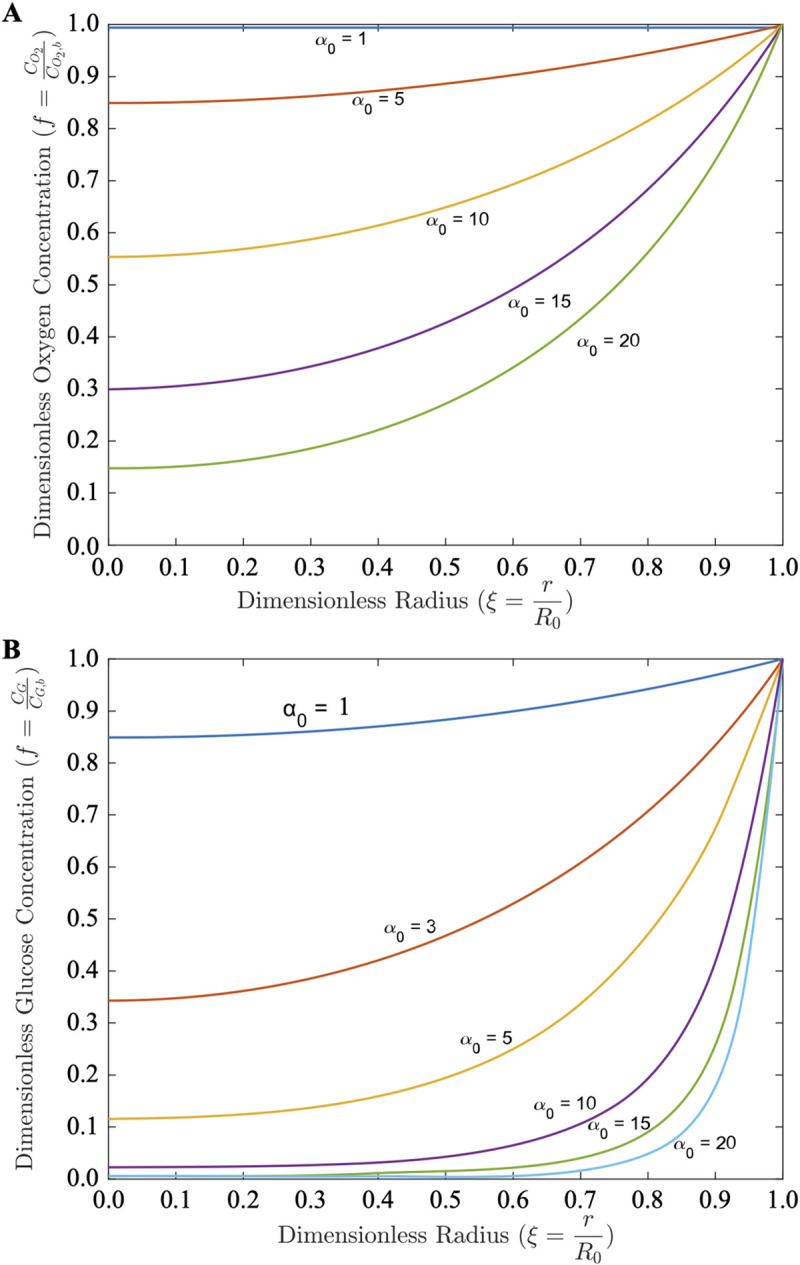
Granuloma oxygen and glucose profile estimates. Dimensionless concentration, *f*, of oxygen (**A**) and glucose (**B**) as a function of dimensionless granuloma radius, *ξ*, for increasing values of dimensionless granuloma size (the modulus *α*_0_).

**Fig 6:**
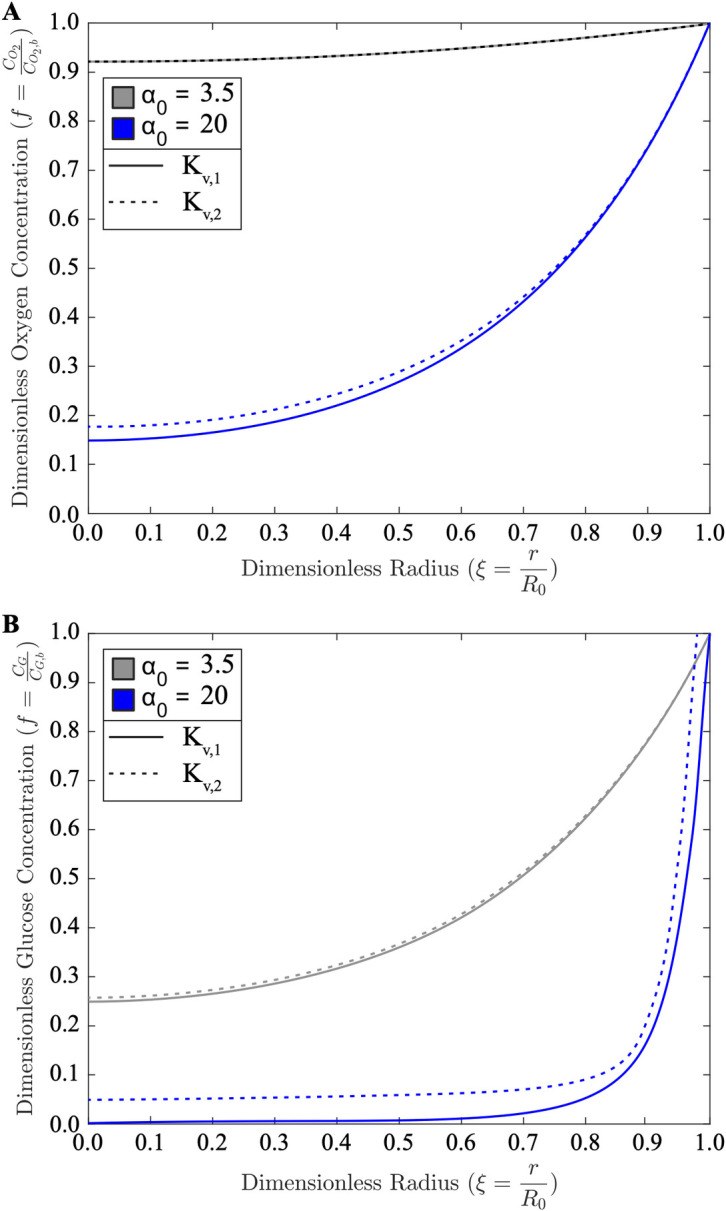
Effect of tissue hydraulic conductivity on oxygen and glucose delivery. (**A**) Oxygen and (**B**) glucose concentration profiles for base case parameter values (Eq S39, see S1 Text) of tissue hydraulic conductivity *K*_*v*_ increased by a factor of 10 for small (*α*_0_ = 3.5) and large (*α*_0_ = 20) granulomas.

**Fig 7:**
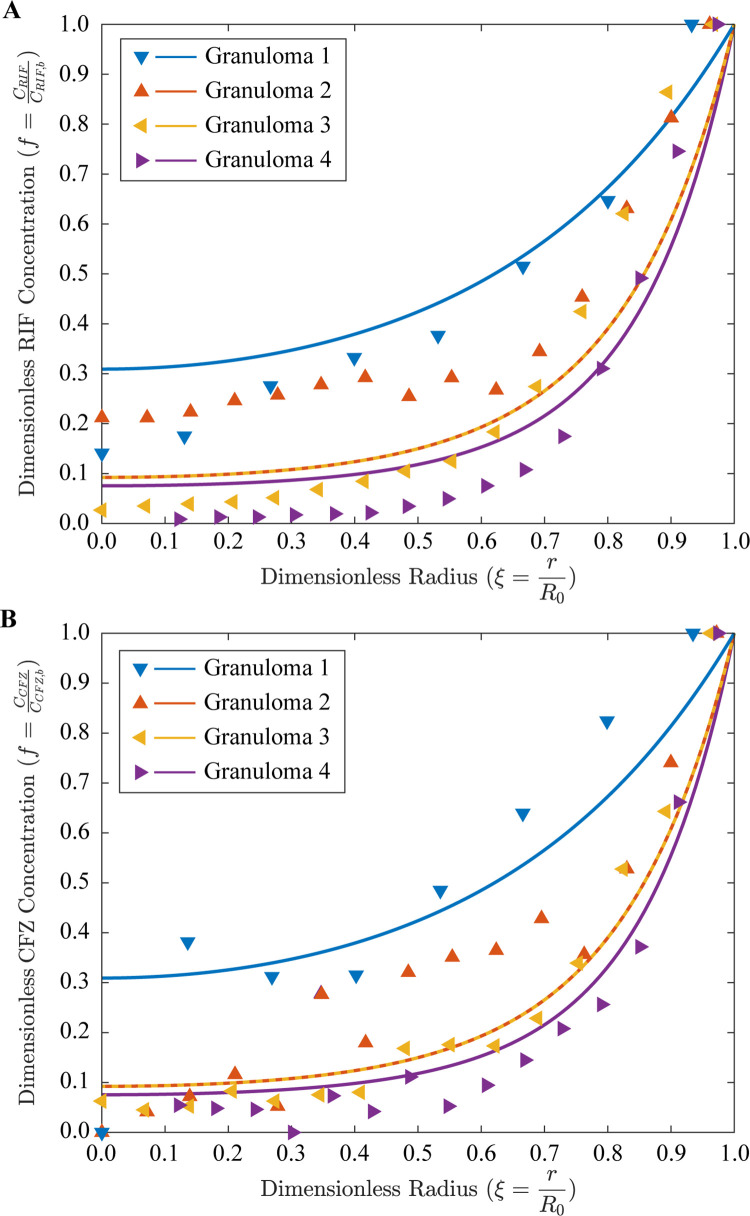
Estimates of drug delivery and comparisons to rifampicin and clofazimine distribution data in experimental granulomas. Dimensionless concentration (lines), *f*, of rifampicin (**A**; RIF) and clofazimine (**B**; CFZ) as a function of dimensionless granuloma radius, *ξ*, for increasing values of dimensionless granuloma size (the modulus *α*_0_) in comparison to experimental data from mouse TB granulomas [17] (dots). The mean squared error (MSE, see Eq S44) between the theoretical and experimental results for rifampicin and clofazimine are 0.012 and 0.010, respectively (see **S2 Table** for all raw data, predicted data, and MSE values).

The description here is limited to the physiological basis (**Fig 1**) and main equations. The complete model derivation, assumptions, boundary conditions, parameter values, analytical solutions, and additional findings are provided in S1 Text. Results were plotted in **Figs 2–7** for IFP (from Eq S30), IFV (from Eq S33), and chemical species concentrations (from Eq S14) from the main equations described below in Model Formulation and Assumptions and in S1 Text, using the built-in function “NDSolve” in Mathematica (Wolfram Research Inc., Champaign, IL).

### Model formulation and assumptions

We assume here that the transcapillary exchange of oxygen is limited to an outer thoroughly vascularized shell [5,15]. The rest of the GME is considered as completely avascular, thus allowing the assumptions of: 1) no extravasation, and 2) only diffusive (and no convective) transport therein (**Fig 1**). (In reality, the blood microvessel density declines more gradually [5], instead of an abrupt transition between well-perfused and inner regions assumed in our shell-core model; see **S1 Fig**). The main equations governing the figures shown in the Results are as follows (see S1 Text for the governing equations and derivation).

Transport of oxygen and other small molecules in an idealized, spherical, vascularized granuloma can be written as a one-dimensional (radial) mass balance in the interstitial space (equivalent to Eq S11 in S1 Text)

$$\underset{\mathrm{Accumulation}}{\underset{︸}{\frac{\partial C_{j}}{\partial t}}}+\underset{\mathrm{Convection}}{\underset{︸}{v_{r}\frac{\partial C_{j}}{\partial r}}}-\underset{\mathrm{Diffusion}}{\underset{︸}{D_{j}^{e}(\frac{2}{r}\frac{\partial C_{j}}{\partial r}+\frac{\partial^{2}C_{j}}{\partial r^{2}})}}=-\underset{\mathrm{Reaction}}{\underset{︸}{\frac{kC_{j}}{1+KC_{j}}}}+\underset{\mathrm{Extravasation}\;\mathrm{and}\;\mathrm{Dilution}}{\underset{︸}{L_{p}a_{v}(p_{v,e}-p_{i})(C_{j,b}-C_{j})}}$$ (Eq I)

Where, for any chemical species *j* (e.g., oxygen, a nutrient, or a drug), its concentration is *C*_*j*_, which varies with respect to time (*t*) and radial location (*r*) within the idealized granuloma (**Fig 1**). Compared to our prior computational model (which only considered diffusion and reaction), we now include convection, extravasation, and dilution terms which we describe in detail here. On the left-hand side: 1) the accumulation term becomes *0* assuming steady-state (i.e., no accumulation of oxygen in the GME), and 2) the convection (second term on the left; *v*_*r*_ is the radial interstitial fluid velocity) and diffusion (third term on the left; *D*_*j*_^*e*^ is the effective diffusion coefficient of species *j* in the interstitial fluid) terms account for interstitial transport of species *j*. On the right-hand side: 3) the reaction term is assumed to follow Michaelis-Menten kinetics of cellular consumption (e.g., of oxygen, where *k* is the first-order rate constant for species consumption and *K* is the inverse of the half-saturation Michaelis-Menten constant) [15], while 4) the last term includes transvascular transport (i.e., extravasation) and plasma dilution (where *L*_*p*_ is the hydraulic conductivity of the membrane-like blood vessel wall, *a*_*v*_ is the volumetric vessel surface area in the tissue, *p*_*ve*_ is the effective vessel pressure, *p*_*i*_ is the interstitial fluid pressure, and *C*_*j*,*b*_ is the bulk concentration of the species *j* in plasma). In dimensionless form (dimensionless terms defined in Eq S12 in S1 Text), the steady-state mass balance (in terms of dimensionless species concentration *f* over dimensionless radial distance *ξ*) reduces to (equivalent to Eq S14 in S1 Text)

$$\frac{d^{2}f}{d\xi^{2}}-\left\{\left({Pe}_{R_{0}}\right)\nu -\frac{2}{\xi }\right\}\frac{df}{d\xi }-\phi^{2}\left(\frac{f}{1+\chi f}\right)+(\alpha^{2}\omega )\varrho (1-f)=0$$ (Eq II)

where *Pe* is the Péclet number, i.e., the ratio of convective to diffusive transport, *ν* is the dimensionless radial interstitial velocity, *ϕ*^2^ is the Thiele modulus, i.e., the ratio of reaction to diffusion rates, *χ* is the dimensionless Michaelis-Menten kinetic factor, *α* is the dimensionless modulus (i.e., granuloma size), *ω* is the dimensionless diffusion rate, and *ϱ* is the relative extravasation/dilution rate.

Explicit expressions for interstitial fluid pressure and velocity derive from the steady-state continuity equation, subject to suitable assumptions and boundary conditions (Eq S14 and S21) as described in the S1 Text. Upon testing multiple perfusion distribution scenarios (**S1–S3 Figs**), the idealized case of uniform perfusion was selected as the final form used here, given the striking similarity of its analytical solutions to the physiological reality of non-uniform perfusion (**S3 Fig**). In dimensionless forms, the radial IFP and IFV are, respectively (equivalent to Eqs S30 and S33 in S1 Text)

$$\psi =1-\varrho =1-\frac{1}{\xi }\frac{\sinh\left(\alpha_{0}\xi \right)}{\sinh\left(\alpha_{0}\right)}$$ (Eq III)

$$\nu =\left(\frac{1}{\alpha_{0}\;\coth\;\alpha_{0}-1}\right)\frac{1}{\xi^{2}}\left\{\frac{(\alpha_{0}\xi )\cosh\left(\alpha_{0}\xi \right)}{\sinh\;\alpha_{0}}-\frac{\sinh\left(\alpha_{0}\xi \right)}{\sinh\;\alpha_{0}}\right\}$$ (Eq IV)

where *ψ* is dimensionless IFP, *ν* is dimensionless IFV, and *α*_0_ is the dimensionless form of the outer granuloma radius (i.e., maximum dimensionless modulus).

### Experimental data and parameter values

To the best of our knowledge, although IFP and IFV are well-studied in cancerous tumors, they have thus far not been theoretically investigated nor experimentally measured in TB granulomas. This means that any corroboration of the theoretical results must depend on corresponding experimental results for tumors. We predict TB granuloma pressure and velocity profiles in the Results, hypothesizing that granuloma properties are similar to those of tumors (Eq S39 and Eq S41 in S1 Text) based on our previous observations of morphological and functional similarities between cancerous tumors and TB granulomas in experiments [5]. Using tumor parameter values, the dimensionless granuloma size (i.e., the modulus *α*) should be in the range of *α*_0_ = 1−15 in our simulations, as a common granuloma diameter in the rabbit model is 2*R*_0_ = 0.5−5 mm (although they can coalesce into larger lesions) [5,16]. We compare these simulations to IFP/IFV data from tumor models [9]. The oxygen consumption Michaelis-Menten parameters are utilized based on our previous work in rabbit TB granulomas [15]. Drug delivery data from mouse models of TB [17] are compared to our simulations, with estimated tissue diffusion coefficients informed by anti-TB drug uptake in rats [18]. All parameters are summarized in **S1 Table** and all raw and predicted drug delivery data and calculated mean square error (MSE) values are in **S2 Table**.

## Results

### Interstitial Fluid Pressure and Velocity Radial Profiles

We and others have experimentally and theoretically investigated the interstitial fluid pressure (IFP) in tumors [8,19–26]. We hypothesize here that there is an analogous IFP rise within TB granulomas, which has not yet been experimentally investigated. Theoretical IFP (**Fig 2A**) and IFV (**Fig 3A**) profiles are shown for varying values of the modulus *α*. IFP rises with distance into the granuloma, while effusive IFV follows an opposite trend and decreases towards the interior of the granuloma. We theorize in analogy to tumors [8] that i) the plasma that leaks out of the abnormal blood vessels as previously described [5] causes this rise in the IFP towards the granuloma core and ii) this is exacerbated by a paucity of functional lymph vessels As IFP rises, the pressure drop declines until it eventually becomes zero. This is given by the Starling equation that accounts for the plasma flux across the microvessel walls between i) the effective vessel pressure (*p*_*v*,*e*_ ≡ *p*_*v*_−*σ*_*S*_(*π*_*v*_−*π*_*i*_)), where *p*_*v*_ is the vascular pressure (mmHg), *π*_*v*_ and *π*_*i*_ are the vascular and interstitial osmotic pressures (mmHg), respectively, and *σ*_*S*_ is the Staverman reflection coefficient) and ii) the IFP (*p*_*i*_, mmHg). This is especially apparent for larger granulomas (**Fig 2A**, *α*_0_>10), where the IFP approaches the effective vessel pressure rapidly with increasing distance towards the core.

As a result of the reducing pressure differential, the effusive (or filtration) flux of plasma, *ϕ*_*B*_, from the blood vessels (bearing oxygen, nutrients, and drugs) declines in the inward radial direction eventually becoming zero. Consequently, at this point, there is no further source for oxygen as well as other nutrients or small molecules/drugs, so that beyond this point the radial flux of these entities is entirely by their diffusion along with any interstitial (albeit compromised) fluid convection. Thus, IFV drops and stagnates in the core; this is especially apparent for large granulomas (**Fig 3A**, *α*_0_>10) where the IFV drops to zero rapidly with increasing distance into the granuloma. At the periphery of the granuloma (*r* → *R*_0_) we may infer that, as in tumors [8], the IFV is directed outwards, which would further impede transport in the GME.

### Model comparisons to experimental IFP/IFV measurements

DiResta et al. measured IFP and IFV in human neuroblastoma tumor models in rats [9]. Our predictive models of IFP (**Fig 2B**) and IFV (**Fig 3B**) align well with these experimental measurements (despite large error bars from the *in-situ* measurements) using a fitted value of *α*_0_ = 4.95, analogous to **S3 Fig**. Thus, the simple analytical solution for the uniform perfusion case is strikingly accurate in predicting *in vivo* IFP/IFV levels, even in large lesions that are presumably non-uniformly perfused and likely contain hypoxic and necrotic regions.

### Convective zone thickness

As depicted schematically in **Fig 1**, convection dominates in the vascularized, well-perfused outer rim of the GME, i.e., the region closest to the normal lung parenchyma. We can estimate the thickness of this rim (Δ = *R*_0_−*R*_*D*_, **Fig 1**) by assuming that within this shell, the pressure gradient becomes small enough that convective velocity approaches 0, representing the turning point at which diffusion dominates (i.e., this can be defined as the limited perfusion velocity; see S1 Text). Utilizing this assumption in Eq IV (equivalent to Eq S33 from the S1 Text), the dimensionless thickness of the convection zone, *λ*_Δ_, is obtained and plotted vs. granuloma size for varying limited perfusion velocities (**Fig 4**). For small granulomas (*α*_0_<5), the majority of the GME is predicted to be well-vascularized; this aligns with experimental observations [5,16]. For 5<*α*_0_<10, the convective zone thickness drastically declines with increasing granuloma size until, at *α*_0_>10, it plateaus. This indicates that for large granulomas, the convection zone occupies less than half of the total mass thickness and the thickness of the convection zone becomes independent of total granuloma size. Utilizing our previously collected data for convective zone thickness from the rabbit TB model [15], in conjunction with theoretical parameter estimates from tumors (Eq S39 in S1 Text), we observe good agreement between our predicted and experimental results.

### Oxygen and glucose concentration profiles

Expanding our previous modeling of avascular granulomas limited to diffusive transport combined with cellular consumption [15], our current model predicts oxygen concentration profiles while accounting for transcapillary exchange, plasma dilution, and interstitial convection and diffusion (**Fig 5A**). As expected, oxygen concentration drops quickly with distance into the granuloma core with increasing granuloma size. For *α*_0_>10, oxygen is predicted to be fully consumed rapidly within the GME; in reality, we know from prior measurements that necrosis emerges due to cell death at oxygen levels nearing–but not reaching–anoxia [5,16]. Similar profiles result from the modeling of glucose transport (**Fig 5B**)–a slightly larger chemical species with poorer diffusivity (**S1 Table**) than molecular oxygen–resulting in more rapid consumption of this nutrient particularly for *α*_0_>10, wherein the central GME is predicted to be devoid of glucose.

### Overcoming interstitial transport barriers

The effect of tissue hydraulic conductivity on oxygen transport can be observed via *in silico* perturbation, whereby the simulated dimensionless oxygen concentration profile within the GME is shown for when the estimated base case parameter of tissue hydraulic conductivity *K*_*v*_ (Eq S39 in S1 Text) is increased by a factor of 10 for small (*α*_0_ = 3.5) and large (*α*_0_ = 20) granulomas (**Fig 6A**). For both small and large granulomas, increasing hydraulic conductivity does not significantly affect oxygen concentration. However, in the large granuloma, the base value hydraulic conductivity value results in the glucose concentration dropping quickly to zero around a dimensionless radius value of 0.5 (**Fig 6B**). This implies that this nutrient is theoretically absent from the central GME under base tissue hydraulic conductivity. Significantly increasing tissue hydraulic conductivity (e.g., by drug treatment), results in glucose not being fully consumed before reaching the granuloma core. Indeed, a paucity of glucose is no longer predicted to emerge even in this very large simulated granuloma with the improvement in hydraulic conductivity, which suggests improved glucose distribution in the granuloma core.

### Model comparisons to experimental drug delivery measurements in granulomas

Finally, this model can be applied not only to oxygen and nutrient delivery, but to anti-TB drug delivery as well. In a recent study [17], Kokesch-Himmelreich et al. measured drug delivery of rifampicin (RIF; molecular weight = 823 g/mol), clofazimine (CFZ; molecular weight = 473 g/mol), and other antibacterial agents using the spatially-resolved matrix-assisted laser desorption/ionization mass spectrometry imaging (MALDI-MSI) method in murine TB granulomas. We extracted and non-dimensionalized the spatially-resolved drug delivery data for RIF and CFZ in four granulomas (all *R*_0_<0.5 mm, *α*_0_<5), from this paper [17]. Strikingly, our simple mass transport model accurately predicts the delivery of these two anti-TB agents, with low MSE values of ~0.01 for both drugs (**Fig 7 and S2 Table**).

## Discussion

A detailed understanding of the drug transport barriers within granulomas can illuminate causes underlying the necessary prolonged treatment for TB, and suggest approaches to alleviate these physiological abnormalities of the GME that hinder transport. Thus, analogous modeling approaches from cancer research are applied here, based on the structural and morphological similarities between TB granulomas and tumors [5] despite the obvious differences between the two disease etiologies. Indeed, common modeling approaches for tumors that have been explored in TB granulomas by us and others include reaction-diffusion [15,27], continuum [28,29], agent-based [30–32], and multiscale [33–35] models, and vascular network/angiogenesis models from tumors [36,37] are likely to be applied to TB granulomas in the future. The approximate analytical solution we obtained previously [15], for the case of diffusion-limited reaction, was able to predict the size of hypoxic and necrotic regions in good agreement with our experimental results from the rabbit TB model. Here, we extended our mathematical framework to include the effect of convective transport within the GME as a result of its abnormal vasculature, and to determine its impact on oxygen, nutrient, and drug delivery.

### Model features, findings, and limitations

Transport phenomena including pressure rise, velocity profile, and interstitial diffusion, are dependent on the structural properties of the GME. We assume that the TB granuloma structure is similar to that of a tumor [21], which is comprised of three regions [5,15]: 1) the vascular region, limited to the periphery and devoid of functional lymphatic vessels; 2) the cellular region containing the cells; and 3) the interstitial (extra-cellular) space that contains an extracellular matrix network that imparts mechanical rigidity, bathed in interstitial fluid. We have shown previously that granuloma vessels are structurally and functionally abnormal, leading to inhomogeneous transport [5]. In tumors, this aberrant vasculature compromises transvascular and interstitial convection due to high vessel permeability and an increase in IFP from plasma leakage and a lack of functional lymphatics. Together, these effects result in an outward convective interstitial velocity that opposes inward transport. By assuming that the GME recapitulates these transvascular and interstitial transport limitations seen in tumors based on our prior experimental evidence [5], the objective of our modeling effort was to predict these parameters and to indicate fruitful directions for future experimental investigations.

We provide here a comprehensive theoretical model of oxygen transport and reaction within a granuloma (or a tumor) that accounts for non-uniform vasculature, transcapillary exchange, plasma dilution, and interstitial convection and diffusion. Three limiting models of vasculature distribution were considered simultaneously for the first time: a model of uniform MVD distribution, a shell-core model, and a non-uniform MVD distribution model. Based on our analyses for IFP and IFV, we can conclude that the simpler case of uniform MVD distribution is adequate for predicting species transport and reaction. In contrast to earlier tumor IFP/IFV models [8,19], our model accounts for plasma dilution (which is often excluded in the literature as negligible). Our mass balance formulation accounts for plasma extravasation from blood vessels not only as a source for oxygen, nutrients, and drugs, but as a diluent as well. Indeed, our model of interstitial transport was found to be in accord with tumor experiments. Predictions of convective zone thickness were found to be in agreement with experimental data from granulomas. The model was also utilized to theoretically investigate the effect of enhanced tissue hydraulic conductivity for overcoming transport barriers. Finally, our model accurately predicted the delivery of two anti-TB agents–rifampicin (first-line therapy) and clofazimine (second-line therapy)–with surprisingly good agreement given the simplicity of the mathematics describing the underlying transport phenomena.

In short, this model sheds light on the limitations of oxygen, nutrient, and drug transport within granulomas and tumors, and how such barriers might be overcome. For example, it predicts an IFP rise towards the granuloma core as a result of the known abnormal vasculature. This in turn results in a reduced or even stagnated IFV, compromising interstitial convective transport and thus depriving the granuloma core–where the bacilli hide–of oxygen, nutrients, and drugs. Our model predicts an absence of glucose in the granuloma core; this warrants experimental confirmation. The persistent bacteria in the hypoxic and necrotic core are able to utilize alternative carbon sources (e.g., lactate) [38]; thus, these metabolic alterations may present targetable vulnerabilities for therapeutic strategies. Modulating the interstitial hydraulic conductivity, e.g., with an agent targeting the interstitial components (such as losartan, a widely-prescribed, safe and inexpensive anti-hypertensive drug [39]), could prove to be an effective host-directed therapeutic strategy to modulate the GME and improve drug delivery and efficacy.

There are some limitations to the model. As described in detail in S1 Text, the non-uniform and/or shell-core models of perfusion better represent the physiological reality of blood vessel distribution in granulomas than the uniform perfusion model. However, the simpler limiting case of uniform perfusion advantageously allows for analytical solutions that 1) do not require artificially defining regions where convection or diffusion dominate, 2) are in good agreement when fitted with the non-uniform perfusion numerical solutions, and, most importantly, 3) accurately predict IFP/IFV experimental data. We also ignore cell membrane transport as a factor in the mass balance equation; however, we account for the cellular reaction of the species (i.e., oxygen consumption). Because we do not have experimental parameter measurements for TB granulomas (e.g., membrane and interstitial hydraulic conductivities) we apply tumor parameter values to the model given the similarities between granulomas and tumors, based on our previous experimental and computational observations regarding shared morphological and functional characteristics of these diseased masses (including vascular density, architecture, and perfusion) [5,15]. We also do not consider variable oxygen/nutrient/drug uptake rates by different types of immune cells, e.g., macrophages vs. T cells, which we have recently demonstrated experimentally can contribute to heterogeneous drug distribution within the GME [40]. Finally, because we do not consider the transport of large molecules here, we ignore any retardation factor. Indeed, because anti-TB agents often bind to proteins such as albumin [41], it may become necessary to model the unbound vs. bound drug fractions in future considerations.

### Future directions

To the best of our knowledge, we provide here the first consideration of convective transport in the GME, and initial predictions for IFP and IFV profiles and their associated consequences in TB granulomas. To further support these findings, it should be experimentally confirmed whether–as in tumors–TB granulomas lack functional lymphatics. It should be noted that the first predictions of tumor IFP/IFV [8] preceded, yet accurately predicted, the first experimental measurements of these parameters by a number of years [42–47]. We posit that future IFP/IFV measurements in TB granulomas will similarly confirm our predictions here. Continued comparison of tumors and TB granulomas via modeling may reveal novel similarities and differences between these two types of masses, particularly with regards to immune state and function. Furthermore, our findings support future testing of host-directed therapies that can modulate the GME to overcome transport barriers and improve treatment outcomes for this virulent and deadly disease. Indeed, following recent efforts by others to computationally optimize the implementation of multiple antibiotic treatments [48], future modeling efforts should provide rational basis for multi-drug dosing and scheduling.

## Supporting information

S1 Text Supporting Information. S1. Species mass balance in granulomas and tumors. S2. Interstitial fluid pressure and velocity profiles. S2.1. Shell-core model of non-uniform perfusion in granulomas. S2.2. Uniform perfusion case. S2.3. Non-uniform perfusion case. S3. Comparison of interstitial perfusion for varying vascular distribution. S3.1. Predictions with the shell-core model. S3.2. Predictions with the uniform vasculature model. S3.3 Comparisons with the non-uniform vessel distribution. S4. Overcoming transport barriers. S5. Convective zone thickness. S6. Mean squared error. Abbreviations, symbols, and terminology.
(DOCX)

S1 Table Parameter values for the mathematical model of transport in TB granulomas. (DOCX)

S2 Table Experimental (Exp.), modeled (Mod.), and differences (Diff) between experimental and modeled data for drug delivery in TB granulomas for CFZ and RIF delivery, with MSE calculated on a per granuloma basis. (DOCX)

S1 Fig Granuloma microvascular density and non-uniform perfusion estimates. Comparison of experimental microvessel density (MVD; dots) in rabbit granulomas [5] versus fitting (lines) via Eq S34 (see S1 Text) for the following parameters: *β* = 1/2; *R*_0_ = 3 mm *L*_*p*_ = 2.8×10^−7^ cm∙mmHg^−1^∙s^−1^, *K*_*v*_ = 4.13×10^−8^ cm^2^∙mmHg^−1^∙s^−1^, *N*_*v*,0_ = 1,000, and *a*_*v*,0_ = 200 cm^2^∙cm^−3^.
(TIFF)

S2 Fig Granuloma IFP estimates from the shell-core model. Predicted dimensionless (**A**) IFP and (**B**) IFP profiles within granulomas for different moduli *α*_0_ and for the shell-core perfusion model, with *ξ*_*D*_ = 0.5.
(TIFF)

S3 Fig Granuloma IFP estimate comparisons from non-uniform, shell-core, and uniform perfusion models. Predicted dimensionless IFP rise within granulomas for the uniform (Eq III), shell-core (Eq S25, see S1 Text), and non-uniform perfusion (Eq S35) models for case of *α*_0_ = 6, with an additional fitted uniform perfusion case for *α*_0_ = 4.1.
(TIFF)

## References

[1] MacNeil A, Glaziou P, Sismanidis C, Date A, Maloney S, Floyd K. Global Epidemiology of Tuberculosis and Progress Toward Meeting Global Targets—Worldwide, 2018. MMWR Morb Mortal Wkly Rep. 2020;69(11):281–5. doi: 10.15585/mmwr.mm6911a2 32191687 PMC7739980

[2] Dartois V. The path of anti-tuberculosis drugs: from blood to lesions to mycobacterial cells. Nature reviews Microbiology. 2014;12(3):159–67. doi: 10.1038/nrmicro3200 24487820 PMC4341982

[3] Jain RK. 1995 Whitaker Lecture: delivery of molecules, particles, and cells to solid tumors. Annals of biomedical engineering. 1996;24(4):457–73. doi: 10.1007/BF02648108 8841721

[4] Dewhirst MW, Secomb TW. Transport of drugs from blood vessels to tumour tissue. Nat Rev Cancer. 2017;17(12):738–50. doi: 10.1038/nrc.2017.93 29123246 PMC6371795

[5] Datta M, Via LE, Kamoun WS, Liu C, Chen W, Seano G, et al. Anti-vascular endothelial growth factor treatment normalizes tuberculosis granuloma vasculature and improves small molecule delivery. Proceedings of the National Academy of Sciences of the United States of America. 2015;112(6):1827–32. doi: 10.1073/pnas.1424563112 25624495 PMC4330784

[6] Jain RK. Antiangiogenesis strategies revisited: from starving tumors to alleviating hypoxia. Cancer Cell. 2014;26(5):605–22. doi: 10.1016/j.ccell.2014.10.006 25517747 PMC4269830

[7] Chauhan VP, Stylianopoulos T, Boucher Y, Jain RK. Delivery of molecular and nanoscale medicine to tumors: transport barriers and strategies. Annual review of chemical and biomolecular engineering. 2011;2:281–98. doi: 10.1146/annurev-chembioeng-061010-114300 22432620

[8] Baxter LT, Jain RK. Transport of fluid and macromolecules in tumors. I. Role of interstitial pressure and convection. Microvascular research. 1989;37(1):77–104. doi: 10.1016/0026-2862(89)90074-5 2646512

[9] DiResta GR, Lee J, Larson SM, Arbit E. Characterization of neuroblastoma xenograft in rat flank. I. Growth, interstitial fluid pressure, and interstitial fluid velocity distribution profiles. Microvascular research. 1993;46(2):158–77. doi: 10.1006/mvre.1993.1044 8246816

[10] Stylianopoulos T, Jain RK. Combining two strategies to improve perfusion and drug delivery in solid tumors. Proceedings of the National Academy of Sciences of the United States of America. 2013;110(46):18632–7. doi: 10.1073/pnas.1318415110 24167277 PMC3832007

[11] Stylianopoulos T, Martin JD, Snuderl M, Mpekris F, Jain SR, Jain RK. Coevolution of solid stress and interstitial fluid pressure in tumors during progression: implications for vascular collapse. Cancer research. 2013;73(13):3833–41. doi: 10.1158/0008-5472.CAN-12-4521 23633490 PMC3702668

[12] Stillman NR, Kovacevic M, Balaz I, Hauert S. In silico modelling of cancer nanomedicine, across scales and transport barriers. Npj Comput Mater. 2020;6(1).

[13] Stylianopoulos T, Munn LL, Jain RK. Reengineering the Physical Microenvironment of Tumors to Improve Drug Delivery and Efficacy: From Mathematical Modeling to Bench to Bedside. Trends Cancer. 2018;4(4):292–319. doi: 10.1016/j.trecan.2018.02.005 29606314 PMC5930008

[14] Stylianopoulos T, Munn LL, Jain RK. Reengineering the Tumor Vasculature: Improving Drug Delivery and Efficacy. Trends Cancer. 2018;4(4):258–9. doi: 10.1016/j.trecan.2018.02.010 29606306 PMC6161778

[15] Datta M, Via LE, Chen W, Baish JW, Xu L, Barry CE, 3rd, et al. Mathematical Model of Oxygen Transport in Tuberculosis Granulomas. Annals of Biomedical Engineering. 2016;44(4):863–72. doi: 10.1007/s10439-015-1415-3 26253038 PMC4795989

[16] Via LE, Lin PL, Ray SM, Carrillo J, Allen SS, Eum SY, et al. Tuberculous granulomas are hypoxic in guinea pigs, rabbits, and nonhuman primates. Infect Immun. 2008;76(6):2333–40. doi: 10.1128/IAI.01515-07 18347040 PMC2423064

[17] Kokesch-Himmelreich J, Treu A, Race AM, Walter K, Holscher C, Rompp A. Do Anti-tuberculosis Drugs Reach Their Target? horizontal line High-Resolution Matrix-Assisted Laser Desorption/Ionization Mass Spectrometry Imaging Provides Information on Drug Penetration into Necrotic Granulomas. Anal Chem. 2022;94(14):5483–92. 35344339 10.1021/acs.analchem.1c03462

[18] Oreilly JR, Corrigan OI, Odriscoll CM. The Effect of Mixed Micellar Systems, Bile-Salt Fatty-Acids, on the Solubility and Intestinal-Absorption of Clofazimine (B663) in the Anesthetized Rat. Int J Pharmaceut. 1994;109(2):147–54.

[19] Arifin DY, Lee KY, Wang CH, Smith KA. Role of convective flow in carmustine delivery to a brain tumor. Pharm Res. 2009;26(10):2289–302. doi: 10.1007/s11095-009-9945-8 19639394

[20] Butler TP, Grantham FH, Gullino PM. Bulk transfer of fluid in the interstitial compartment of mammary tumors. Cancer research. 1975;35(11 Pt 1):3084–8. 1182701

[21] Jain RK. Transport of molecules in the tumor interstitium: a review. Cancer research. 1987;47(12):3039–51. 3555767

[22] Liu LJ, Brown SL, Ewing JR, Schlesinger M. Phenomenological model of interstitial fluid pressure in a solid tumor. Physical review E, Statistical, nonlinear, and soft matter physics. 2011;84(2 Pt 1):021919. doi: 10.1103/PhysRevE.84.021919 21929031 PMC3533446

[23] Sefidgar M, Soltani M, Raahemifar K, Sadeghi M, Bazmara H, Bazargan M, et al. Numerical modeling of drug delivery in a dynamic solid tumor microvasculature. Microvascular research. 2015;99:43–56. doi: 10.1016/j.mvr.2015.02.007 25724978

[24] Soltani M, Chen P. Numerical modeling of fluid flow in solid tumors. PloS one. 2011;6(6):e20344. doi: 10.1371/journal.pone.0020344 21673952 PMC3108959

[25] Swabb EA, Wei J, Gullino PM. Diffusion and convection in normal and neoplastic tissues. Cancer research. 1974;34(10):2814–22. 4369924

[26] Swartz MA, Fleury ME. Interstitial flow and its effects in soft tissues. Annual review of biomedical engineering. 2007;9:229–56. doi: 10.1146/annurev.bioeng.9.060906.151850 17459001

[27] Catala M, Prats C, Lopez D, Cardona PJ, Alonso S. A reaction-diffusion model to understand granulomas formation inside secondary lobule during tuberculosis infection. PloS One. 2020;15(9):e0239289. doi: 10.1371/journal.pone.0239289 32936814 PMC7494083

[28] Hao W, Schlesinger LS, Friedman A. Modeling Granulomas in Response to Infection in the Lung. PloS One. 2016;11(3):e0148738. doi: 10.1371/journal.pone.0148738 26986986 PMC4795641

[29] Fallahi-Sichani M, Schaller MA, Kirschner DE, Kunkel SL, Linderman JJ. Identification of key processes that control tumor necrosis factor availability in a tuberculosis granuloma. PLoS Comput Biol. 2010;6(5):e1000778. doi: 10.1371/journal.pcbi.1000778 20463877 PMC2865521

[30] Sershen CL, Plimpton SJ, May EE. A method for modeling oxygen diffusion in an agent-based model with application to host-pathogen infection. Annu Int Conf IEEE Eng Med Biol Soc. 2014;2014:306–9. doi: 10.1109/EMBC.2014.6943590 25569958

[31] Pienaar E, Cilfone NA, Lin PL, Dartois V, Mattila JT, Butler JR, et al. A computational tool integrating host immunity with antibiotic dynamics to study tuberculosis treatment. J Theor Biol. 2015;367:166–79. doi: 10.1016/j.jtbi.2014.11.021 25497475 PMC4332617

[32] Pienaar E, Dartois V, Linderman JJ, Kirschner DE. In silico evaluation and exploration of antibiotic tuberculosis treatment regimens. BMC Syst Biol. 2015;9:79. doi: 10.1186/s12918-015-0221-8 26578235 PMC4650854

[33] Pienaar E, Matern WM, Linderman JJ, Bader JS, Kirschner DE. Multiscale Model of Mycobacterium tuberculosis Infection Maps Metabolite and Gene Perturbations to Granuloma Sterilization Predictions. Infect Immun. 2016;84(5):1650–69. doi: 10.1128/IAI.01438-15 26975995 PMC4862722

[34] Sershen CL, Plimpton SJ, May EE. Oxygen Modulates the Effectiveness of Granuloma Mediated Host Response to Mycobacterium tuberculosis: A Multiscale Computational Biology Approach. Front Cell Infect Microbiol. 2016;6:6. doi: 10.3389/fcimb.2016.00006 26913242 PMC4753379

[35] Bowness R, Chaplain MAJ, Powathil GG, Gillespie SH. Modelling the effects of bacterial cell state and spatial location on tuberculosis treatment: Insights from a hybrid multiscale cellular automaton model. J Theor Biol. 2018;446:87–100. doi: 10.1016/j.jtbi.2018.03.006 29524441 PMC5901892

[36] Hormuth DA 2nd, Phillips CM, Wu C, Lima E, Lorenzo G, Jha PK, et al. Biologically-Based Mathematical Modeling of Tumor Vasculature and Angiogenesis via Time-Resolved Imaging Data. Cancers (Basel). 2021;13(12). doi: 10.3390/cancers13123008 34208448 PMC8234316

[37] Baish JW, Stylianopoulos T, Lanning RM, Kamoun WS, Fukumura D, Munn LL, et al. Scaling rules for diffusive drug delivery in tumor and normal tissues. Proceedings of the National Academy of Sciences of the United States of America. 2011;108(5):1799–803. doi: 10.1073/pnas.1018154108 21224417 PMC3033252

[38] Kiran D, Basaraba RJ. Lactate Metabolism and Signaling in Tuberculosis and Cancer: A Comparative Review. Front Cell Infect Microbiol. 2021;11:624607. doi: 10.3389/fcimb.2021.624607 33718271 PMC7952876

[39] Datta M, Chatterjee S, Perez EM, Gritsch S, Roberge S, Duquette M, et al. Losartan controls immune checkpoint blocker-induced edema and improves survival in glioblastoma mouse models. Proceedings of the National Academy of Sciences of the United States of America. 2023;120(6):e2219199120. doi: 10.1073/pnas.2219199120 36724255 PMC9963691

[40] Blanc L, Daudelin IB, Podell BK, Chen PY, Zimmerman M, Martinot AJ, et al. High-resolution mapping of fluoroquinolones in TB rabbit lesions reveals specific distribution in immune cell types. Elife. 2018;7. doi: 10.7554/eLife.41115 30427309 PMC6249001

[41] Alghamdi WA, Al-Shaer MH, Peloquin CA. Protein Binding of First-Line Antituberculosis Drugs. Antimicrob Agents Chemother. 2018;62(7). doi: 10.1128/AAC.00641-18 29735566 PMC6021678

[42] Boucher Y, Jain RK. Microvascular pressure is the principal driving force for interstitial hypertension in solid tumors: implications for vascular collapse. Cancer research. 1992;52(18):5110–4. 1516068

[43] Boucher Y, Kirkwood JM, Opacic D, Desantis M, Jain RK. Interstitial hypertension in superficial metastatic melanomas in humans. Cancer Research. 1991;51(24):6691–4. 1742743

[44] Boucher Y, Leunig M, Jain RK. Tumor angiogenesis and interstitial hypertension. Cancer Research. 1996;56(18):4264–6. 8797602

[45] Boucher Y, Salehi H, Witwer B, Harsh GRt, Jain RK. Interstitial fluid pressure in intracranial tumours in patients and in rodents. Br J Cancer. 1997;75(6):829–36. doi: 10.1038/bjc.1997.148 9062403 PMC2063404

[46] Less JR, Posner MC, Boucher Y, Borochovitz D, Wolmark N, Jain RK. Interstitial hypertension in human breast and colorectal tumors. Cancer Research. 1992;52(22):6371–4. 1423283

[47] Roh HD, Boucher Y, Kalnicki S, Buchsbaum R, Bloomer WD, Jain RK. Interstitial hypertension in carcinoma of uterine cervix in patients: possible correlation with tumor oxygenation and radiation response. Cancer Research. 1991;51(24):6695–8. 1742744

[48] Cicchese JM, Pienaar E, Kirschner DE, Linderman JJ. Applying optimization algorithms to tuberculosis antibiotic treatment regimens. Cell Mol Bioeng. 2017;10(6):523–35. doi: 10.1007/s12195-017-0507-6 29276546 PMC5737793

