# Supplementary material for: Mathematical model of oxygen, nutrient, and drug transport in tuberculosis granulomas
Source: PLoS Comput Biol. 2024 Feb 9;20(2):e1011847. doi: 10.1371/journal.pcbi.1011847 (PMC10883541; doi:10.1371/journal.pcbi.1011847)
Supplement: S1 Text — S1. Species mass balance in granulomas and tumors. S2. Interstitial fluid pressure and velocity profiles. S2.1. Shell-core model of non-uniform perfusion in granulomas. S2.2. Uniform perfusion case. S2.3. Non-uniform perfusion case. S3. Comparison of interstitial perfusion for varying vascular distribution. S3.1. Predictions with the shell-core model. S3.2. Predictions with the uniform vasculature model. S3.3 Comparisons with the non-uniform vessel distribution. S4. Overcoming transport barriers. S5. Convective zone thickness. S6. Mean squared error. Abbreviations, symbols, and terminology. (DOCX) [file pcbi.1011847.s001.docx]

S1 Text

**Mathematical Model of Oxygen, Nutrient, and Drug Transport in Tuberculosis Granulomas**

Meenal Datta, McCarthy Kennedy, Saeed Siri, Laura E. Via, James W. Baish, Lei Xu, Veronique Dartois, Clifton E. Barry 3^rd^, and Rakesh K. Jain

**S1. Species Mass Balance in Granulomas and Tumors**

A blood-borne molecular nutrient or drug species undergoes the following sequential transport steps (1, 2) within a granuloma or a tumor: 1) extravasation across the capillary wall; 2) transport through the interstitial space via convection and diffusion; 3) transport across a cell membrane; and 4) metabolic reaction within the cell. We neglect the cell membrane transfer resistance here, but account for the cellular reaction of a species. Further, for larger molecules such as monoclonal antibodies or proteins, not considered here, there is specific or non-specific binding (3).

We adopt a coarse-grained continuum approach, and start with the molar form of unsteady-state mass balance of species *j* (oxygen, nutrient, or a drug) within the granuloma or tumor (4), i.e.,

| $\frac{\partial C_{j}}{\partial t}+\text{∇}\cdot\text{N}_{j}=R_{j}$ | (Eq.S1) |
| --- | --- |

where $R_{j}$ is the volumetric source term for species *j*. The flux of a species *j*, in general, contains both convective and diffusive terms, which for dilute solutions is of the form (4)

| $\text{N}_{j}=\mathcal{v}C_{j}-D_{j}^{e}\text{∇}C_{j}$ | (Eq.S2) |
| --- | --- |

The effective diffusivity $D_{j}^{e}$of a molecule in the interstitial region may be estimated from its molecular diffusion coefficient $D_{j}$ via the relation $D_{j}^{e}=(\varepsilon/\tau)D_{j}$ (5), where $\varepsilon$ is the volume fraction (or void fraction) of interstitial space among the cells, and $\tau$ is the so-called tortuosity factor, accounting for the tortuous diffusion paths. This is based on the assumption that the transport is limited to the interstitial domain. This is not true for molecules such as oxygen, which might well diffuse through the cellular region as well. Further, the tortuosity factor in turn can be related to the interstitial volume fraction. For example, the random pore model suggests $\tau=1/\varepsilon$. More generally, $\tau=1/\varepsilon^{q}$, where, *q* = 0.5 refers to the so-called Bruggeman exponent. Other factors besides the structure, e.g., partition coefficient, or solubility, can also affect the effective diffusion coefficient of a molecule (1, 6). Finally, since we are not considering large molecules, we ignore any retardation factor (1).

Combining the last two relations and carrying out the indicated differential operation

| $\frac{\partial C_{j}}{\partial t}+\mathcal{v}\cdot{\text{∇}C}_{j}+C_{j}\text{∇}\cdot\mathcal{v}-D_{j}^{e}\text{∇}^{2}C_{j}=R_{j}$ | (Eq.S3) |
| --- | --- |

The convective term itself is, thus, comprised of two parts: 1) the usual *convective* term $\mathcal{v}\cdot{\text{∇}C}_{j}$, and 2) the *dilution* term $C_{j}\text{∇}\cdot\mathcal{v}$ containing the divergence of fluid velocity, $\text{∇}\cdot\mathcal{v}$. For transport in incompressible fluids (liquids), the second term is typically zero, since from the equation of continuity $\text{∇}\cdot\mathcal{v}=0$ (4). However, for a granuloma or a tumor comprising of blood and lymphatic vessels that provide a net source/sink term for the fluid, the equation of continuity, i.e., overall mass balance, instead takes the form (3, 5)

| $\text{∇}\cdot\mathcal{v}=\varphi_{B}(\mathbf{r})-\varphi_{L}(\mathbf{r})$ | (Eq.S4) |
| --- | --- |

where $\varphi_{B}\left( \mathbf{r} \right) {(\text{s}}^{-1})$ is the position ($\mathbf{r}$) dependent interstitial fluid source term i.e., the net volumetric rate of plasma extravasated by blood vessels per unit volume of the granuloma or tumor, while $\varphi_{L}(\mathbf{r})$ is the corresponding term for the drainage of the interstitial fluid by lymphatics. In biological tissues these terms represent the rate of plasma flow from blood vessels into the interstitial space, and from interstitial space into the lymph vessels, respectively (see Reference (5) for schematic representations).

The distribution of these vessels is well-known to vary with location **r**, within the tumor (3), and has also been shown to similarly vary within granulomas (8). In the case of the diffusion-limited region (denoted by subscript *D*), where there is a complete absence of such vessels, Eq.S4 reduces to the common form of the equation of continuity, $\text{∇}\cdot\mathcal{v}=0$.

Combining the last two relations, the mass balance in granulomas or tumors takes the form

| $\frac{\partial C_{j}}{\partial t}+\mathcal{v}\cdot{\boldsymbol{\nabla}C}_{j}-D_{j}^{e}\nabla^{2}C_{j}=R_{j}-C_{j}\mathbf{(}\varphi_{B}-\varphi_{L}\mathbf{)}$ | (Eq.S5) |
| --- | --- |

Further, in the above, the net rate of the species “source” term $R_{j}$ within the tissue is a sum of two terms: 1) that due to reaction, and 2) that due to the fluid source term

| $R_{j}=-\frac{kC_{j}}{1+KC_{j}}+\varphi_{B}C_{j,b}$ | (Eq.S6) |
| --- | --- |

where the metabolic reaction rate term is assumed of the Michaelis-Menten (MM) form, as is common for many cellular reactions, with a negative sign denoting “consumption,” rather than generation, while the source term of species *j* from the blood vessels is $\varphi_{B}C_{j,b}$, where $C_{j,b}$ is the concentration of the species *j* in the bulk plasma. The MM kinetics appropriately reduce to first-order when $KC_{j}\ll1$, with a pseudo-first order rate constant $k$, while it reduces to zero-order for the case when $KC_{j}\gg1$, when kinetic rate is equal to $k/K$ (9). In terms of the more traditional form, the MM kinetics are written as: $r_{j}=V_{max}C_{j}/(K_{M}+C_{j})$, where the MM parameters, $V_{max}=k/K$, and the MM constant, $K_{M}=1/K$. In our recent publication (9), further, only the first term in Eq.S6 was included, as applicable to the diffusion-dominated quiescent inner region where there are no blood vessels. This assumption is relaxed here.

Combining the last two relations

| $\frac{\partial C_{j}}{\partial t}+\mathcal{v}\cdot{\text{∇}C}_{j}-D_{j}^{e}\text{∇}^{2}C_{j}=-\frac{kC_{j}}{1+KC_{j}}+\varphi_{B}\left( C_{j,b}-C_{j} \right)\mathbf{+}\varphi_{L}C_{j}$ | (Eq.S7) |
| --- | --- |

which in one-dimensional (radial) form

| $\frac{\partial C_{j}}{\partial t}+\mathcal{v}_{r}\frac{\partial C_{j}}{\partial r}-D_{j}^{e}\frac{1}{r^{2}}\frac{\partial}{\partial r}\left( r^{2}\frac{\partial C_{j}}{\partial r} \right)=-\frac{kC_{j}}{1+KC_{j}}+\varphi_{B}\left( C_{j,b}-C_{j} \right)\mathbf{+}\varphi_{L}C_{j}$ | (Eq.S8) |
| --- | --- |

This formulation is a bit different from the corresponding mass balance equations in the literature, because it accounts for the effect of extravasation not only as a source for species *j* but also as a diluent, latter by virtue of the use of equation of continuity, Eq.S4.

In solid tumors, further, there are few functional lymph vessels, and so that $\varphi_{L}\to0$ (1). Even if this were not completely true, certainly $\varphi_{B}\gg\varphi_{L}$, as blood vessels are leakier in tumors, so that $\varphi_{L}$ may be neglected. We make the same assumption here for TB granulomas. Further, carrying out the indicated differentiation in the Laplacian term, the species mass balance reduces to

| $\frac{\partial C_{j}}{\partial t}+\mathcal{v}_{r}\frac{\partial C_{j}}{\partial r}-D_{j}^{e}\left( \frac{2}{r}\frac{\partial C_{j}}{\partial r}+\frac{\partial^{2}C_{j}}{\partial r^{2}} \right)=-\frac{kC_{j}}{1+KC_{j}}+\varphi_{B}\left( C_{j,b}-C_{j} \right)$ | (Eq.S9) |
| --- | --- |

The plasma flux across the microvessel walls is further given by the Starling equation (3, 5)

| $\varphi_{B}\equiv\frac{J_{v}}{V}=L_{p}a_{v}\left\{ \left( p_{v}-p_{i} \right)-\sigma_{S}{(\pi}_{v}-\pi_{i}) \right\}=L_{p}a_{v}(p_{v,e}-p_{i})$ | (Eq.S10) |
| --- | --- |

where $J_{v}/V$ is the volumetric flow rate out of the vasculature per unit volume of tissue, $L_{p}$ is the membrane hydraulic conductivity of vessels (cm^4^/s·mmHg) within the granuloma, $a_{v}(r)$ is the surface area of the vessels/volume of tissue (cm^2^/cm^3^), i.e., *S*/*V*, which varies with location *r*, owing to the heterogeneous nature of blood vessel distribution in granulomas or tumors. For example, $a_{v}=N_{v}(\pi d_{v})l_{v}$, where $N_{v}(r)$ is the number density of microvessels/volume, and $d_{v}$ and $l_{v}$ are their average diameter and length.

Further, $p_{v}$ and $p_{i}$ are the vascular and interstitial pressures (mmHg), respectively, while $\pi_{v}$ and $\pi_{i}$ are the corresponding osmotic pressures (mmHg), and $\sigma_{S}$ is the Staverman reflection coefficient. In other words, the driving force is the hydrostatic pressure difference minus the osmotic pressure difference between the inside of the vessels and the interstitial space in tissue. For a single solute, for instance, the osmotic pressure, $\pi=-(RT/\bar{V}_{j})\text{ln}a_{j}$, i.e., it depends on the species activity or concentration. Further, in Eq.S10, we have defined an effective vessel pressure, $p_{v,e}\equiv p_{v}-\sigma_{S}{(\pi}_{v}-\pi_{i})$.

Combining the last two equations

| $\underset{\text{Accumulation}}{\underbrace{\frac{\partial C_{j}}{\partial t}}}+\underset{\text{Convection}}{\underbrace{\mathcal{v}_{r}\frac{\partial C_{j}}{\partial r}}}-\underset{\text{Diffusion}}{\underbrace{D_{j}^{e}\left( \frac{2}{r}\frac{\partial C_{j}}{\partial r}+\frac{\partial^{2}C_{j}}{\partial r^{2}} \right)}}=-\underset{\text{Reaction}}{\underbrace{\frac{kC_{j}}{1+KC_{j}}}}+\underset{\text{Extravasation and Dilution}}{\underbrace{L_{p}a_{v}(p_{v,e}-p_{i})\left( C_{j,b}-C_{j} \right)}}$ | (Eq.S11) |
| --- | --- |

where the different transport (convection and diffusion), accumulation, and source (reaction and extravasation) terms are as indicated. This may be compared to the mass balance equation of our previous work (9), that assumes: 1) that the flux is by diffusion only (i.e., $\mathcal{v}_{r}=0$, or no convection), and 2) that the last term on the right, accounting for transcapillary extravasation and plasma dilution, is zero as there are no blood vessels in the diffusion-limited region. In other words, it involves only diffusion and reaction, as in a conventional catalyst particle, the convection and the fluid source term being assumed zero.

For this, we next non-dimensionalize this mass balance equation using the following dimensionless variables

| $\xi\equiv\frac{r}{R_{0}}; \psi\equiv\frac{p_{i}}{p_{v,e}} ; \varrho\equiv1-\frac{p_{i}}{p_{v,e}}; f\equiv\frac{C_{j}}{C_{j,b}} ; \nu\equiv\frac{\mathcal{v}_{r}}{\mathcal{v}_{R_{0}}} ;{Pe}_{R_{0}}\equiv\frac{{\mathcal{v}_{R_{0}}R}_{0}}{D_{j}^{e}};$ $\phi\equiv R_{0}\sqrt{\frac{k}{D_{j}^{e}}} ; \alpha\left( \xi\right)\equiv R_{0}\sqrt{\frac{L_{p}a_{v}\left( \xi\right)}{K_{v}}} ; \chi\equiv KC_{j,b} ; \tau\equiv\left( \frac{D_{j}^{e}}{R_{0}^{2}} \right)t ; \omega\equiv\frac{K_{v}p_{v,e}}{D_{j}^{e}} ;$ | (Eq.S12) |
| --- | --- |

It is noteworthy that for given membrane and tissue properties, the modulus $\alpha\propto R_{0}$, since the square root term is independent of granuloma/tumor size, i.e., a bigger modulus means a larger granuloma/tumor. In other words, the modulus $\alpha$ may be roughly considered as dimensionless radius of granuloma/tumor.

In dimensionless form, the mass balance, thus, the unsteady-state mass balance for species *j* is

| $\frac{\partial f}{\partial\tau}+\left( {Pe}_{R_{0}} \right)\nu\frac{\partial f}{\partial\xi}-\left( \frac{2}{\xi}\frac{\partial f}{\partial\xi}+\frac{\partial^{2}f}{\partial\xi^{2}} \right)=-\phi^{2}\frac{f}{1+\chi f}+(\alpha^{2}\omega)\varrho\left( 1-f \right)$ | (Eq.S13) |
| --- | --- |

Besides the dimensionless concentration, $f(\xi)$, this equation involves the convection term on the left that is a function of $\xi$ via the dimensionless velocity $\nu(\xi)$, as well as the extravasation term, i.e., the last term on the right, also a function of $\xi$ via the dimensionless pressure difference $\varrho(\xi)$. Thus, other relations are needed for $\nu(\xi)$ and $\varrho(\xi)$, as discussed next.

For the steady-state case, the species mass balance reduces to

| $\frac{d^{2}f}{d\xi^{2}}-\left\{ \left( {Pe}_{R_{0}} \right)\nu-\frac{2}{\xi} \right\}\frac{df}{d\xi}-\phi^{2}\left( \frac{f}{1+\chi f} \right)+(\alpha^{2}\omega)\varrho\left( 1-f \right)\boldsymbol{=}0$ | (Eq.S14) |
| --- | --- |

subject to the boundary conditions

| $\begin{matrix} \text{B.C. 1}: & \text{at} \xi=1 & f=1 \\ \text{B.C. 2}: & \text{at} \xi=0 & \frac{df}{d\xi}=0 \end{matrix}$ |  |
| --- | --- |

The expressions for the dimensionless interstitial velocity $\nu(\xi)$ and the volumetric effusion term, $\Omega(\xi)\equiv$ $\alpha^{2}\omega\varrho(\xi)$, are developed in the next section.

**S2. Interstitial Fluid Pressure and Velocity Profiles**

We and others have experimentally and theoretically investigated the interstitial fluid pressure (IFP) in tumors (1, 3, 7, 10-15). It is hypothesized that there is an analogous IFP rise within TB granulomas, not yet experimentally investigated, as modeled below (5).

The steady-state equation of continuity, or fluid mass balance, Eq.S4 provides $\boldsymbol{\nabla}\cdot\mathcal{v}=\varphi_{B}(\mathbf{r})-\varphi_{L}(\mathbf{r})$, which is combined with the constitutive equation for viscous flow in porous media, namely D’Arcy’s law, $\mathcal{v} =-{(B}_{0}/\mu)\boldsymbol{\nabla}p_{i}$, normally written in the tumor literature as (15)

| $\mathcal{v} =-K_{v}\boldsymbol{\nabla}p_{i}$ | (Eq.S15) |
| --- | --- |

where $K_{v}\equiv(B_{0}/\mu)$, is the so-called hydraulic conductivity of the tumor or granuloma, while $B_{0}$ is the D’Arcy permeability, to provide

| $-K_{v}\nabla^{2}p_{i} =\varphi_{B}-\varphi_{L}$ | (Eq.S16) |
| --- | --- |

Using the Laplacian for a one-dimensional transport in a spherical shaped granuloma, Eq.S16 reduces to

| $-K_{v}\frac{1}{r^{2}}\frac{d}{dr}\left( r^{2}\frac{dp_{i}}{dr} \right)=\varphi_{B}-\varphi_{L}$ | (Eq.S17) |
| --- | --- |

Assuming as above, that either $\varphi_{L}\to0$, or that $\varphi_{B}\gg\varphi_{L}$, for the TB granulomas and combining Eqns. (Eq.S10) and (Eq.S17)

| $-K_{v}\frac{1}{r^{2}}\frac{d}{dr}\left( r^{2}\frac{dp_{i}}{dr} \right)-L_{p}a_{v}(p_{v,e}-p_{i})=0$ | (Eq.S18) |
| --- | --- |

where recall that $a_{v}=a_{v}(\xi)$. This can be written in the dimensionless form

| $\frac{1}{\xi^{2}}\frac{d}{d\xi}\left( \xi^{2}\frac{d\varrho}{d\xi} \right)-\alpha^{2}\varrho=0$ | (Eq.S19) |
| --- | --- |

where recall that the dimensionless interstitial pressure *difference*, $\varrho\equiv(p_{v,e}-p_{i})/p_{v,e}=1-\psi$, and the dimensionless interstitial pressure, $\psi\equiv p_{i}/p_{v,e}$.

In general, as indicated above, the microvessel surface area per unit volume also varies with location, i.e., $a_{v}=a_{v}(\xi)$, so that the parameter $\alpha=\alpha(\xi)$. We consider three different cases for the vasculature architecture as described below:

- 1. the case of uniform perfusion throughout the granuloma/tumor, wherein $a_{v}=a_{v,0}=$ constant, i.e., independent of $\xi$;
  2. the case of non-uniform perfusion described via step change (3), wherein the diffusion-limited hypoxic core has no vessels ($a_{v}=0$), while the outer shell of the granuloma/tumor is fully vascularized ($a_{v}=a_{v,0}$), i.e., the shell-core model, and
  3. the case of non-uniform perfusion with a realistic variation in microvessel density (MVD) distribution, $N_{v}=N_{v}(\xi$).

The first two cases allow analytical solution for the IFP and IFV, while the last requires a numerical solution. We consider below the more general Case 2 first, from which Case 1 derives as a limiting case.

**S2.1. Shell-Core Model of Non-uniform Perfusion in Granulomas**

Adapting the approach of Baxter and Jain (1990) for tumors to granulomas, it is assumed here that in the region $R_{D}<r<R_{0}$, $a_{v}=a_{v,0}$, while in the region $r<R_{D}$, $a_{v}=0$. Thus, the modulus $\alpha=\alpha_{0}$ in the shell region, defined as

| $\alpha_{0}\equiv R_{0}\sqrt{\frac{L_{p}a_{v,0}}{K_{v}}}$ | (Eq.S20) |
| --- | --- |

which represents a ratio of the interstitial resistance to vascular resistance to IF flow.

The momentum balance equation is subject to the following boundary conditions (16):

| $\text{BC 1: at} \xi=1, \varrho=1,\text{ i.e.,} \psi=0 \left( \text{or} p_{i}=0 \right),$  $\text{BC 2: at} \xi=\xi_{D}, \frac{d\varrho}{d\xi}=\text{0;} \varrho=\varrho_{D}\text{.}$ | (Eq.S21) |
| --- | --- |

where the first boundary condition specifies that the pressure at the periphery, $p_{i}=0$, while the second represents no flow, or pressure gradient, along with continuity of pressure at the diffusion-limited core radius $R_{D}$.

The general solution to Eq.S19, which is of the same form as that for molecular diffusion and first order reaction in granuloma (9), is

| $\varrho=\frac{C_{1}}{\xi}\cosh\left( \alpha_{0}\xi\right)+\frac{C_{2}}{\xi}\sinh\left( \alpha_{0}\xi\right)$ | (Eq.S22) |
| --- | --- |

which upon differentiating provides

| $\frac{d\varrho}{d\xi}=\frac{C_{1}}{\xi^{2}}\left\{ \alpha_{0}\xi\sinh\left( \alpha_{0}\xi\right)-\cosh\left( \alpha_{0}\xi\right) \right\}+\frac{C_{2}}{\xi^{2}}\left\{ \alpha_{0}\xi\cosh\left( \alpha_{0}\xi\right)-\sinh\left( \alpha_{0}\xi\right) \right\}$ | (Eq.S23) |
| --- | --- |

Using B.C. 2 in Eq.S23 while using B.C. 1 in Eq.S22, and solving the resulting two equations simultaneously for the two unknown constants of integration $C_{1}$ and $C_{2}$, followed by some simplification, provides

| $C_{1}=-\frac{\sinh\left( \alpha_{0}\xi_{D} \right)-\alpha_{0}\xi_{D}\cosh\left( \alpha_{0}\xi_{D} \right)}{\sinh\left[ \alpha_{0}\left( 1-\xi_{D} \right) \right]+\alpha_{0}\xi_{D}\cosh\left[ \alpha_{0}\left( 1-\xi_{D} \right) \right]}$  $C_{2}=-\frac{\alpha_{0}\xi_{D}\sinh\left( \alpha_{0}\xi_{D} \right)-\cosh\left( \alpha_{0}\xi_{D} \right)}{\sinh[\alpha_{0}(1-\xi_{D})]+\alpha_{0}\xi_{D}\cosh[\alpha_{0}(1-\xi_{D})]}$ | (Eq.S24) |
| --- | --- |

Using these in Eq.S22 and simplifying further, the dimensionless pressure differential (16)

| $\varrho(\xi)=\frac{1}{\xi}\left\{ \frac{\sinh[\alpha_{0}(\xi-\xi_{D})]+\alpha_{0}\xi_{D}\cosh[\alpha_{0}(\xi-\xi_{D})]}{\sinh[\alpha_{0}(1-\xi_{D})]+\alpha_{0}\xi_{D}\cosh[\alpha_{0}(1-\xi_{D})]} \right\}$ | (Eq.S25) |
| --- | --- |

where we have used the identities, $\sinh\left( u-v \right)=\sinh u\cosh v-\cosh u\sinh v$ and $\cosh\left( u-v \right)=\cosh u\cosh v-\sinh u\sinh v$. These results are applicable to the shell region, i.e., for $\xi_{D}\leq\xi\leq1$. The dimensionless pressure differential in the non-vascularized core of the particle is obtained by setting $\xi= \xi_{D}$ in Eq.S25.

Further, taking the derivative of the dimensionless pressure gradient, and combining it with the 1-dimensional Darcy’s law $\mathcal{v}_{r}=-K_{v}(dp_{i}/dr)=K_{v}p_{v,e}/R_{0}(d\varrho/d\xi)$, provides the velocity profile within a granuloma

| $\mathcal{v}_{r}=\frac{K_{v}p_{v,e}}{R_{0}}\frac{1}{\xi^{2}}\left\{ \frac{\alpha_{0}(\xi-\xi_{D})\cosh[\alpha_{0}(\xi-\xi_{D})]-\left( 1-\alpha_{0}^{2}\xi_{D}\xi\right)\sinh\left[ \alpha_{0}\left( \xi-\xi_{D} \right) \right]}{\sinh[\alpha_{0}(1-\xi_{D})]+\alpha_{0}\xi_{D}\cosh[\alpha_{0}(1-\xi_{D})]} \right\}$ | (Eq.S26) |
| --- | --- |

which when evaluated at the granuloma surface ($\xi=1$), yields the efflux velocity at the surface

| $\mathcal{v}_{R_{0}}=\left( \frac{K_{v}p_{v,e}}{R_{0}} \right)\left\{ \frac{\alpha_{0}(1-\xi_{D})\cosh[\alpha_{0}(1-\xi_{D})]-\left( 1-\alpha_{0}^{2}\xi_{D} \right)\sinh\left[ \alpha_{0}\left( 1-\xi_{D} \right) \right]}{\sinh[\alpha_{0}(1-\xi_{D})]+\alpha_{0}\xi_{D}\cosh[\alpha_{0}(1-\xi_{D})]} \right\}$ | (Eq.S27) |
| --- | --- |

The surface effusion velocity could alternately be determined as follows. From the Gauss-Ostrogradskii divergence theorem (4), $\int^{V} ( \boldsymbol{\nabla}\cdot\mathcal{v})dV=\int^{S} (\mathcal{v}\cdot\boldsymbol{n})dS$, and using Eq.S4 for the divergence of velocity in this, along with Eq.S10 for the volumetric efflux $\varphi_{B}$, there results

| $\mathcal{v}_{R_{0}}=\left( \frac{K_{v}p_{v,e}}{R_{0}} \right)\int_{\xi_{D}}^{1} (\alpha^{2}\varrho)\xi^{2}d\xi$ | (Eq.S28) |
| --- | --- |

Finally, assuming $\alpha=\alpha_{0}$, using Eq.S25 for $\varrho$ and integrating, Eq.S27 results.

From Eq.S26 and Eq.S27, the dimensionless velocity,$\nu\equiv\mathcal{v}_{r}/\mathcal{v}_{R_{0}}$ ,

| $\nu=\frac{1}{\xi^{2}}\left\{ \frac{\alpha_{0}(\xi-\xi_{D})\cosh[\alpha_{0}(\xi-\xi_{D})]-\left( 1-\alpha_{0}^{2}\xi_{D}\xi\right)\sinh\left[ \alpha_{0}\left( \xi-\xi_{D} \right) \right]}{\alpha_{0}(1-\xi_{D})\cosh[\alpha_{0}(1-\xi_{D})]-\left( 1-\alpha_{0}^{2}\xi_{D} \right)\sinh\left[ \alpha_{0}\left( 1-\xi_{D} \right) \right]} \right\}$ | (Eq.S29) |
| --- | --- |

The above algebraic relations for the dimensionless pressure difference $\varrho$, Eq.S25, and the dimensionless IF velocity $\nu$, Eq.S29, can be utilized in the steady-state species *j* mass balance differential equation, Eq.S14. In other words, Eq.S14, Eq.S25, and Eq.S29 together form a set of differential-algebraic equations that can be solved simultaneously numerically to compute the concentration profile of a molecule within a granuloma. We have already solved the concentration profile, Eq.S14, for the case of no convective flow ($\nu=0$) as well as no source term ($\Omega=0$), where we also provided analytical solutions for the two limiting cases of first- and zero-order kinetics (9). Our analysis here extends our initial results to the more general case to include both convective transport ($\nu\neq0$) as well as a source term ($\Omega\neq0$).

**S2.2. Uniform Perfusion Case**

For the case when there is no hypoxic core, $\xi_{D}\to0$, i.e., the granuloma is uniformly perfused throughout, then the corresponding relations may be obtained by simply setting $\xi_{D}=0$ in the above. Thus, from Eq.S25, the dimensionless pressure rise within the granuloma for the case of uniform perfusion (5)

| $\psi=1-\varrho=1-\frac{1}{\xi}\frac{\sinh\left( \alpha_{0}\xi\right)}{\sinh\left( \alpha_{0} \right)}$ | (Eq.S30) |
| --- | --- |

The radial velocity profile from Eq.S26

| $\mathcal{v}_{r}=\left( \frac{K_{v}p_{v,e}}{R_{0}} \right)\frac{1}{\xi^{2}}\left\{ \frac{\left( \alpha_{0}\xi\right)\cosh\left( \alpha_{0}\xi\right)}{\sinh\alpha_{0}}-\frac{\sinh\left( \alpha_{0}\xi\right)}{\sinh\alpha_{0}} \right\}$ | (Eq.S31) |
| --- | --- |

while the efflux velocity at the granuloma surface, from Eq.S27 with $\xi_{D}=0$

| $\mathcal{v}_{R_{0}}=\left( \frac{K_{v}p_{v,e}}{R_{0}} \right)\left( \alpha_{0}\coth\alpha_{0}-1 \right)$ | (Eq.S32) |
| --- | --- |

so that the dimensionless velocity profile takes the form (5)

| $\nu=\left( \frac{1}{\alpha_{0}\coth\alpha_{0}-1} \right)\frac{1}{\xi^{2}}\left\{ \frac{\left( \alpha_{0}\xi\right)\cosh\left( \alpha_{0}\xi\right)}{\sinh\alpha_{0}}-\frac{\sinh\left( \alpha_{0}\xi\right)}{\sinh\alpha_{0}} \right\}$ | (Eq.S33) |
| --- | --- |

These simpler alternate relations for the volumetric fluid effusion and the dimensionless IFV applicable for the case of uniform vasculature can next be utilized in the species *j* mass balance, Eq.S14, to obtain concentration profile within a granuloma or a tumor.

**S2.3. Non-uniform Perfusion Case**

In the above shell-core model, we considered the case where the granuloma/tumor was assumed to be fully vascularized outside of the hypoxic core, while there was no perfusion within it. This is not physically realistic, as the microvessel density (MVD) distribution does not change so abruptly, but rather more gradually. In fact, the MVD distribution in a granuloma has been measured by us (8), as shown in **Figure S1**.

Clearly the blood vessels are restricted to the granuloma periphery and tend to be absent from the central region, as we found previously (8), but this change happens gradually. In fact, it might be argued that the MVD distribution appears to follow the pressure difference distribution, i.e., the extravasation driving force, $\varrho=1-\psi$. As a result, in analogy with Eq.S30, we might simply assume the MVD distribution

| $\frac{N_{v}(\xi)}{N_{v,0}}=\frac{a_{v}(\xi)}{a_{v,0}}=\frac{\alpha(\xi)}{\alpha_{0}}=\frac{1}{\xi}\frac{\sinh\left( \beta\alpha_{0}\xi\right)}{\sinh\left( {\beta\alpha}_{0} \right)}$ | (Eq.S34) |
| --- | --- |

Where $\beta\sim1/2$ is a fitted constant. This would be used in both momentum balance, Eq.S19, and mass balance, Eq.S14.

It is apparent from **Figure S1** that the agreement is good. There might, in fact, be physiological reasons for this. Thus, we might surmise that functional blood vessels are not present beyond the point where $p_{i}\to p_{v,e}$, since the plasma can no longer flow out of the blood vessels. Clearly, in this case, analytical solution for the pressure profile is not possible, and Eq.S19 must be solved numerically, which, when combined with Eq.S34, may be written as

| $\left( \frac{2}{\xi}\frac{d\varrho}{d\xi}+\frac{d^{2}\varrho}{d\xi^{2}} \right)-\alpha_{0}^{2}\left\{ \frac{1}{\xi}\frac{\sinh\left( \beta\alpha_{0}\xi\right)}{\sinh\left( \beta\alpha_{0} \right)} \right\}\varrho=0$ | (Eq.S35) |
| --- | --- |

A numerical solution of this provides the pressure differential profile within the granuloma. This is next related to the velocity profile via

| $\nu=\frac{1}{\mathcal{v}_{R_{0}}}\left( \frac{K_{v}p_{v,e}}{R_{0}} \right)\left( \frac{d\varrho}{d\xi} \right)$ | (Eq.S36) |
| --- | --- |

where from Eq.S28 and Eq.S34

| $\mathcal{v}_{R_{0}}=\left( \frac{K_{v}p_{v,e}}{R_{0}} \right)\alpha_{0}^{2}\int_{0}^{1} \left\{ \frac{\sinh\left( \beta\alpha_{0}\xi\right)}{\sinh\left( \beta\alpha_{0} \right)} \right\}^{2}\varrho d\xi$ | (Eq.S37) |
| --- | --- |

where $\varrho$ is obtained from a solution to Eq.S35.

Alternately, we can write the dimensionless velocity as

| $\nu=\left( \frac{d\varrho}{d\xi} \right)/{\left( \frac{d\varrho}{d\xi} \right)_{\xi=1}}$ | (Eq.S38) |
| --- | --- |

The last two ODEs, for $\varrho$ (Eq.S35) and for $\nu$ (Eq.S36 or Eq.S38), along with Eq.S14, the ODE representing the mass balance of species *j*, represent a system of three differential equations that need to be solved simultaneously in order to numerically obtain the pressure difference, velocity, and species *j* concentration profiles within the granuloma or the tumor. This is then the most general case considered here, wherein few simplifying assumptions have been made.

**S3. Comparison of Interstitial Perfusion for Varying Vascular Distribution**

Because IFP and IFV have not yet been measured experimentally in granuloma vessels, we first predict these profiles assuming that granulomas and tumors share certain physiological parameters. Thus, in combination with oxygen consumption parameters values from our previous work (9), we use the following parameter values (provided from tumors (1, 3, 5, 12-14)):

| $L_{p}=2.8\times{10}^{-7} \text{cm∙}\text{mmHg}^{-1}\text{∙}\text{s}^{-1}$; $a_{v,0}=200 \text{cm}^{2}\text{∙}\text{cm}^{-3};K_{v}=4.13\times{10}^{-8} \text{cm}^{2}\text{∙}\text{mmHg}^{-1}\text{∙}\text{s}^{-1}$ | (Eq.S39) |
| --- | --- |

Assuming, that these values are a good first estimate for granulomas as well,

$$\sqrt{\frac{L_{p}a_{v,0}}{K_{v}}}=\sqrt{\frac{2.8\times{10}^{-7}\times200}{4.13\times{10}^{-8}}}=36.82 \text{cm}^{-1}$$

Thus, for ${2R}_{0}=0.5-5$ mm, a common size of granulomas observed in rabbits (though they can be larger if multiple lesions coalesce) (8, 17), this provides the range of the modulus $\alpha_{0}=1-15$, which would hence be of the primary interest for granulomas.

**S3.1. Predictions with the Shell Core Model** ($\boldsymbol{\xi}_{\boldsymbol{D}}\boldsymbol{>0}$)

We provide figures (**Figure S2**) of dimensionless pressure rise and velocity profile (Eq.S7) for the case of $\xi_{D}=0.5$ and different values of $\alpha_{0}$ based on the use of Eqs. (Eq.S25) – (Eq.S29). These expressions are applicable to the shell region, i.e., for $\xi_{D}\leq\xi\leq1$. For the hypoxic core, the dimensionless pressure differential is obtained by setting $\xi= \xi_{D}$ in Eq.S25, providing

| $\psi_{0}=1-\varrho_{0}=1-\frac{\alpha_{0}}{\sinh[\alpha_{0}(1-\xi_{D})]+\alpha_{0}\xi_{D}\cosh[\alpha_{0}(1-\xi_{D})]}$ | (Eq.S40) |
| --- | --- |

As **Figure S2A** shows, the pressure rises sharply in the shell and then levels out at the value indicated by Eq.S40. In fact. for $\alpha_{0}>10$, the interstitial pressure in the particle core attains the value in the effective vascular pressure.

**Figure S2B** plots the resulting effusion velocity as a function of radial distance in the shell. The velocity in the core is zero, due to the absence of any pressure gradient therein. It is interesting that for small modulus $\alpha_{0}$, the velocity profile has a slight convexity to it. In fact, it is possible that the small $\alpha_{0}$ values ($0<\alpha_{0}<5$) are inconsistent with the assumptions inherent in the shell-core model, as for small $\alpha_{0}$ , the hypoxic core is unlikely to exist.

**S3.2. Predictions with the Uniform Vasculature Model**  ($\boldsymbol{\xi}_{\boldsymbol{D}}\boldsymbol{\to0}$)

The case of uniform vasculature is actually consistent with smaller values of the modulus $\alpha_{0}$ ($0<\alpha_{0}<5$), which recall is rather a dimensionless particle radius. Nonetheless, the computations for the case of uniform vasculature are provided in for the pressure rise (**Figure 2**) and the velocity profile (**Figure 3**) for a variety of the values of the modulus $\alpha_{0}$.

It is evident that the maximum pressure rises at a given value of $\alpha_{0}$ is higher for the case of uniform perfusion rather than for the case of shell perfusion. The velocity profiles (**Figure 3**) are also more reasonable for smaller values of $\alpha_{0}$ than that in **Figure S2B** based on assuming perfusion limited to a shell.

On the other hand, for the case of larger modulus $\alpha_{0}$ ($\alpha_{0}>7$), the difference in the predictions of the two models are small. In summary, we can surmise that for smaller values of the modulus $\alpha_{0}$ (smaller particles), the uniform perfusion model is a better approximation, while for the case of larger particle sizes and $\alpha_{0}$, the shell-core model is more physically realistic, but even the uniform perfusion model is adequate. In short, the simpler uniform perfusion model is adequate in general.

It is further seen from **Figures 2** and **3** that when flow resistance of the interstitial space is smaller (large $K_{v}$) than the flow resistance of the vessel walls, or when the granuloma particle size is small, i.e., when $\alpha_{0}\leq1$, the pressure rise within the granuloma is small, and the convective velocity of the plasma leaving the vessels is linear all the way to the particle center, so that the entire granuloma is well-perfused and there is no hypoxic ring or necrotic core, i.e., transport of a solute is not limited by diffusion.

On the other hand, when flow resistance of the interstitial space is higher (smaller $K_{v}$) than the flow resistance of the vessel walls, or when the granuloma particle size is large, $\alpha_{0}\gg1$, and the IFP rise is rapid, and within a short distance $\Delta$ of the particle surface, $\psi\to1$ or $p_{i}\to p_{v,e}$. Further, in this case, the convective velocity of the plasma $\mathcal{v}_{r}\to0$ for $\xi<\xi_{D}$, after which convective transport of oxygen/solute comes to a halt, so that the oxygen and other nutrients must diffuse thereafter, resulting in a hypoxic ring, as well as a necrotic core inside the granuloma. Thus, Swabb et al. (1974) concluded that for small molecules, e.g., oxygen, glucose, and amino acids, the transport via diffusive mechanism is dominant. On the other hand, larger molecules such as antibodies are largely dependent on convective transport.

**S3.3. Comparisons with the Non-uniform Vessel Distribution**

The uniform and shell-core model microvessel distributions discussed above are two limiting cases of a more realistic and gradual vessel distribution of the kind discussed above. We next explore how the predictions of pressure rise, e.g., for this case are in comparison to the above two limiting distributions.

Based on the numerical integration of Eq.S35, **Figure S3** provides a comparison of the pressure rise for the case of a particle with MVD distribution as described by Eq.S34 for the case of $\alpha_{0}=6$, which is rather an intermediate value in the range of interest ($0<\alpha_{0}<15$). The case of uniformly distributed vessels Eq.S30 overpredicts the pressure rise in comparison, while the shell-core distribution can provide a reasonable estimate provided the thickness of the well-perused shell is used a s a fitted parameter. Thus, in **Figure S3**, the value of $\xi_{D}=0.48$ provides a good approximation. For that matter, the simple uniform distribution model can also provide a good estimate of the pressure profile, provided the modulus $\alpha_{0}$ is used as a fitted parameter. Thus, the use of $\alpha_{0}=4.1$ provides a pressure rise profile (dotted line) that adequately mimics that resulting from the case of non-uniformly distributed vessels (Eq.S35). In summary, the uniform MVD distribution model would be adequate in most cases for a prediction of the pressure rise, and consequently, the effusion velocity distribution while it has the advantage of simple explicit expressions.

**S4. Overcoming Transport Barriers**

To theoretically investigate the effect of improving delivery (e.g., of oxygen, nutrients, or drugs) by improving the tissue hydraulic conductivity of the tumors or granulomas, we retain as the base case the parameters used above, namely, those in Eq.S39, along with the following typical values:

| $p_{v,e}=20 \text{mmHg}; R_{0}=2.5mm;k=0.01 \text{s}^{-1};D_{j}^{e}=1\times{10}^{-6} {\text{cm}^{2}\text{ s}}^{-1}$ | (Eq.S41) |
| --- | --- |

The first-order rate constant and the effective diffusion coefficient are typical of nutrient of the size of glucose (18), rather than a smaller molecule such as oxygen. Larger molecules (>50,000 Da), e.g., antibodies, are also of interest where a diffusion retardation factor (14) may be needed to describe diffusion in the interstitial space.

Further, as discussed above, the simpler explicit expressions describing the IFP and IFV within a granuloma or tumor for the case of uniform MVD distribution are adequate. Consequently, even though the other cases discussed above, namely, the abrupt shell-core model, or the more gradually declining MVD distribution described by the fitted model, may be more realistic, in what follows, we will simply adopt the uniform MVD distribution model (Eq.S30 and Eq.S33) for use in the steady-state species mass balance relation, Eq.S14.

**Figure 5B** provides the simulated species distribution profile within a tumor or a granuloma based on the above parameters: 1) as the base case for the hydraulic conductivity provided in Eq.S39 ($K_{v}=4.13\times{10}^{-8} \text{cm}^{2}\text{∙}\text{mmHg}^{-1}\text{∙}\text{s}^{-1}$) and 2) the case of improved hydraulic conductivity by a factor of 10. For the parameters adopted, this means the following dimensionless parameters: a Thiele modulus, $\phi=25$; the modulus characteristic of the vessel to tissue hydraulic conductivity, $\alpha_{0}=9.2$; and the parameter $\omega=0.83$. Additionally, for the base case, the surface effusion velocity from Eq.S32, $\mathcal{v}_{R_{0}}=0.27 \mu\text{m}.\text{s}^{-1}$, so that the Peclet number at the surface, ${Pe}_{R_{0}}=6.78$.

The hydraulic conductivity $K_{v}$ can, in fact, be described in terms of tissue structural parameters (15). For example (5), by visualizing a porous medium as a bundle of capillaries of radius *a*, and using the Poiseuille equation for laminar capillary flow, $K_{v}=(\varepsilon/\tau)(a^{2}/8\mu)$, where $\mu$ is the interstitial fluid viscosity. Other factors affecting hydraulic conductivity have been discussed (1).

**S5. Convective Zone Thickness**

The convective zone thickness $\Delta$ of the outer well-perfused rim, where the dimensionless concentration $f\to1$, can be estimated from a numerical solution of the mass balance equation. We are interested in approximately determining the thickness of this zone, where convective transport dominates. It is necessary to select a criterion for this, e.g., at a location when the pressure gradient becomes small enough so that convective velocity → 0 (at the edge of the diffusive, non-vascularized zone, *R*_D_, **Figure 1**), before diffusion becomes the dominant mode of transport. For this, a reasonable estimated Peclet number can be assumed, e.g., ${Pe}_{D}\ll1$. Then from the definition of the Peclet number, one can obtain the corresponding velocity, i.e.,

| $\mathcal{v}_{D}={Pe}_{D}\left( \frac{D_{\text{O}_{2}}^{e}}{R_{0}} \right)$ | (Eq.S42) |
| --- | --- |

For example, for the case of oxygen, with $D_{\text{O}_{2}}^{e}=2.5\times{10}^{-5}$ cm^2^/s, and $R_{0}=0.25$ cm, and assuming ${Pe}_{D}=0.01$, the limiting perfusion velocity, $\mathcal{v}_{D}=1\times{10}^{-6}$ cm/s. Thus, the dimensionless, limiting perfusion velocity for determining the thickness $\Delta$, $\nu_{D}\equiv\mathcal{v}_{D}/\mathcal{v}_{R_{0}}=1\times{10}^{-6}/1.36\times{10}^{-5}=0.073$.

In other words, from Eq.S33 for the uniform MVD distribution case, we can determine the corresponding dimensionless convective zone thickness $\lambda_{\Delta}=1-\xi_{D}$, where $\xi_{D}\equiv R_{D}/R_{0}$, for an assumed of say $\nu_{D}\approx0.001-0.1$, i.e., by solving the following

| $\nu_{D}-\left( \frac{1}{\alpha_{0}\coth\alpha_{0}-1} \right)\left\{ \frac{\alpha_{0}}{\xi_{D}}\frac{\cosh\left( \alpha_{0}\xi_{D} \right)}{\sinh\left( \alpha_{0} \right)}-\frac{1}{\xi_{D}^{2}}\frac{\sinh\left( \alpha_{0}\xi_{D} \right)}{\sinh\left( \alpha_{0} \right)} \right\}=0$ | (Eq.S43) |
| --- | --- |

**S6. Mean Squared Error (MSE)**

The agreement between the experimental drug delivery data and the predicted values from the model can be assessed via the MSE metric, which is reported for each granuloma and drug in **S2 Table.docx**. We calculate MSE as (Eq. S44)

| $MSE=\frac{1}{n}\sum_{i=1}^{n} {(Y-Y^{'})}^{2}$ | (Eq.S44) |
| --- | --- |

Where *Y* represents actual experimental values each measured at i^th^ observed values, *Y’* represents the predicted values, i is and *n* is the total number of data points for each granuloma. A lower MSE indicates a better fit of model predictions to observed data.

**Abbreviations, symbols, and terminology**

| *Abbreviations*: |  |
| --- | --- |
| B.C. | Boundary condition |
| CFZ | Clofazamine |
| Da | Dalton |
| GME | Granuloma microenvironment |
| HDT | Host-directed therapy |
| IFP | Interstitial fluid pressure |
| IFV | Interstitial fluid velocity |
| MM  MSE | Michaelis-Menten  Mean squared error |
| MVD | Microvascular density |
| RIF | Rifampicin |
| ODE | Ordinary differential equation |
| TME | Tumor microenvironment |
| *Roman Symbols:* |  |
| $a_{v}$ | surface area of the vessels/volume of tissue (cm^2^/cm^3^) |
| $B_{0}$ | D’Arcy permeability (cm^-2^) |
| $C_{1,2}$ | Constants of integration |
| $C_{j}$ | Concentration of chemical species *j* (i.e., where *j* is oxygen, a nutrient [e.g., glucose], or a drug) (mol/L) |
| $C_{G}$ | concentration of glucose (mol/cm^3^) |
| $C_{G,b}$ | constant bulk concentration of glucose in the perfused region (mol/L) |
| $C_{O_{2}}$ | concentration of oxygen (mol/cm^3^) |
| $C_{O_{2,b}}$ | constant bulk concentration of oxygen in the perfused region (mol/L) |
| $D_{CFZ,RIF}$ | effective diffusion coefficient of CFZ and RIF in the interstitial fluid (mm^2^/s) |
| $D_{j}^{e}$ | the effective diffusion coefficient of chemical species *j* in the interstitial fluid (mm^2^/s) |
| $d_{v}$ | average microvessel diameter (mm) |
| $f$ | dimensionless concentration of oxygen |
| $J_{v}$ | vascular volumetric flow rate (mm3/s) |
| $k$ | first-order rate constant for oxygen consumption (s^-1^) |
| $K$ | inverse of the half-saturation Michaelis-Menten constant (L/mol) |
| $K_{M}$ | half-saturation constant of the Michaelis-Menten equation (mol/L) |
| $K_{v}$ | hydraulic conductivity coefficient (mm/s) |
| $L_{p}$ | membrane hydraulic conductivity of blood vessels (mm^4^/s-mmHg) |
| $l_{v}$  *n* | average microvessel length (mm)  number of drug delivery points gathered experimentally per granuloma |
| $N_{v}$ | microvessel density (number of microvessels/mm^2^) |
| $p_{i}$ | interstitial fluid pressure (mmHg) |
| $p_{v,e}$ | effective vessel pressure (mmHg) |
| ${Pe}_{r}$  ${Pe}_{R_{0}}$ | Péclet number for radial transport  Péclet number at the granuloma-lung interface (at *r = R_0_*) |
| ${Pe}_{\Delta}$ | Péclet number below which diffusion dominates |
| $q$ | Bruggeman exponent |
| $r$ | radial position in the granuloma (mm) |
| $\Delta r$ | radial thickness over which the shell-balance is performed (mm) |
| $R_{0}$ | radius of the granuloma (mm) |
| $R_{D}$ | radius of diffusive region (mm) |
| $R_{j}$ | volumetric source term for chemical species *j* |
| $v_{r}$ | radial interstitial fluid velocity (mm/s) |
| $v_{\Delta}$ | radial velocity associated with ${Pe}_{\Delta}$ (mm/s) |
| $V$ | granuloma/tumor volume (mm3) |
| $V_{max}$  *Y*  *Y’* | maximum rate of respiration (mol/mm3 s)  experimental (observed) value of normalized drug delivery in a TB granuloma  modeled (predicted) value of normalized drug delivery in a TB granuloma |
| *Greek Symbols:* |  |
| $\alpha_{0}$ | dimensionless modulus (i.e., granuloma/tumor size) |
| $\chi$ | dimensionless Michaelis-Menten kinetic factor |
| $\Delta$ | thickness of well-perfused layer (mm) |
| $\varepsilon$ | volume fraction |
| $\phi$ | Thiele modulus |
| $\varphi_{B}$ | volumetric rate of oxygen delivery from the vasculature into the tissue (mm^3^/s) |
| $\varphi_{L}$ | volumetric flow rate of plasma to the lymph vessels (mm^3^/s) |
| $\kappa$ | ratio of the first-order rate constant for oxygen consumption to the half-saturation constant of the Michaelis-Menten equation (mol/Ls) |
| $\lambda_{\Delta}$ | dimensionless well-perfused layer thickness |
| $\pi_{i}$ | interstitial oncotic pressure (mmHg) |
| $\pi_{v}$ | vessel oncotic pressure (mmHg) |
| $\varrho$ | dimensionless pressure difference |
| $\sigma_{S}$ | solute (Staverman) reflection coefficient |
| $\tau$ | tortuosity factor |
| $\nu$ | dimensionless IFV |
| $\xi$ | dimensionless radial depth |
| $\psi$ | dimensionless IFP |

**References:**

1. Jain RK. Transport of molecules in the tumor interstitium: a review. Cancer research. 1987;47(12):3039-51.

2. Dartois V. The path of anti-tuberculosis drugs: from blood to lesions to mycobacterial cells. Nature reviews Microbiology. 2014;12(3):159-67.

3. Baxter LT, Jain RK. Transport of fluid and macromolecules in tumors. I. Role of interstitial pressure and convection. Microvascular research. 1989;37(1):77-104.

4. Bird RB, Stewart WE, Lightfoot EN. Transport Phenomena: John Wiley & Sons; 2007.

5. Truskey GA, Yuan F, Katz DF. Transport phenomena in biological systems. Upper Saddle River, NJ: Pearson/Prentice Hall; 2004.

6. Freyer JP, Sutherland RM. Determination of diffusion constants for metabolites in multicell tumor spheroids. Advances in experimental medicine and biology. 1983;159:463-75.

7. Arifin DY, Lee KY, Wang CH, Smith KA. Role of convective flow in carmustine delivery to a brain tumor. Pharm Res. 2009;26(10):2289-302.

8. Datta M, Via LE, Kamoun WS, Liu C, Chen W, Seano G, et al. Anti-vascular endothelial growth factor treatment normalizes tuberculosis granuloma vasculature and improves small molecule delivery. Proceedings of the National Academy of Sciences of the United States of America. 2015;112(6):1827-32.

9. Datta M, Via LE, Chen W, Baish JW, Xu L, Barry CE, 3rd, et al. Mathematical Model of Oxygen Transport in Tuberculosis Granulomas. Annals of biomedical engineering. 2016;44(4):863-72.

10. Butler TP, Grantham FH, Gullino PM. Bulk transfer of fluid in the interstitial compartment of mammary tumors. Cancer research. 1975;35(11 Pt 1):3084-8.

11. Liu LJ, Brown SL, Ewing JR, Schlesinger M. Phenomenological model of interstitial fluid pressure in a solid tumor. Physical review E, Statistical, nonlinear, and soft matter physics. 2011;84(2 Pt 1):021919.

12. Sefidgar M, Soltani M, Raahemifar K, Sadeghi M, Bazmara H, Bazargan M, et al. Numerical modeling of drug delivery in a dynamic solid tumor microvasculature. Microvascular research. 2015;99:43-56.

13. Soltani M, Chen P. Numerical modeling of fluid flow in solid tumors. PloS one. 2011;6(6):e20344.

14. Swabb EA, Wei J, Gullino PM. Diffusion and convection in normal and neoplastic tissues. Cancer research. 1974;34(10):2814-22.

15. Swartz MA, Fleury ME. Interstitial flow and its effects in soft tissues. Annual review of biomedical engineering. 2007;9:229-56.

16. Baxter LT, Jain RK. Transport of fluid and macromolecules in tumors. II. Role of heterogeneous perfusion and lymphatics. Microvascular research. 1990;40(2):246-63.

17. Via LE, Lin PL, Ray SM, Carrillo J, Allen SS, Eum SY, et al. Tuberculous granulomas are hypoxic in guinea pigs, rabbits, and nonhuman primates. Infect Immun. 2008;76(6):2333-40.

18. Panteli JT, Forbes NS. Engineered bacteria detect spatial profiles in glucose concentration within solid tumor cell masses. Biotechnology and bioengineering. 2016;113(11):2474-84.

19. Oreilly JR, Corrigan OI, Odriscoll CM. The Effect of Mixed Micellar Systems, Bile-Salt Fatty-Acids, on the Solubility and Intestinal-Absorption of Clofazimine (B663) in the Anesthetized Rat. Int J Pharmaceut. 1994;109(2):147-54.
