# Supplementary material for: Mathematical model of oxygen, nutrient, and drug transport in tuberculosis granulomas
Source: PLoS Comput Biol. 2024 Feb 9;20(2):e1011847. doi: 10.1371/journal.pcbi.1011847 (PMC10883541; doi:10.1371/journal.pcbi.1011847)
Supplement: S1 Table — (DOCX) [file pcbi.1011847.s002.docx]

**Table S1.** Parameter values for the mathematical model of transport in TB granulomas.

| Parameter | Value | Units | Reference |
| --- | --- | --- | --- |
| *a_v,0_* | 200 | cm^2^/cm^3^ | (3, 16) |
| $\text{C}_{\text{O}_{\text{2}}\text{,}\text{b}}\text{}$ | 8.04 x 10^-8^ | mol O_2_/cm^3^ | (9) |
| $\text{D}_{\text{G}}^{\text{e}}$ | 1 x 10^-6^ | cm^2^/s | (5) |
| $\text{D}_{\text{O}_{\text{2}}}^{\text{e}}$ | 2.5 x 10^-5^ | cm^2^/s | (9) |
| $\text{D}_{\text{CFZ/RIF}}^{\text{e}}$ | 1 x 10^-7^ | cm^2^/s | (19) |
| $\text{k}_{\text{O}_{\text{2}}}\text{}$ | 1.09 | s^-1^ | (9) |
| *K_v_* | 4.13 x 10^-8^ | cm^2^/(mmHg-s) | (3, 16) |
| *L_p_* | 2.8 x 10^-7^ | cm^2^/(mmHg-s) | (3, 16) |
| $\text{p}_{\text{v,e}}$ | 20 | mmHg | (5) |
