# Supplementary material for: Mathematical model of oxygen, nutrient, and drug transport in tuberculosis granulomas
Source: PLoS Comput Biol. 2024 Feb 9;20(2):e1011847. doi: 10.1371/journal.pcbi.1011847 (PMC10883541; doi:10.1371/journal.pcbi.1011847)
Supplement: S2 Table — (DOCX) [file pcbi.1011847.s003.docx]

**Table S2.**  Experimental (Exp.), modeled (Mod.), and differences (Diff) between experimental and modeled data for drug delivery in TB granulomas for CFZ and RIF delivery, with MSE calculated on a per granuloma basis.

| RIF, Granuloma 1 | | | RIF, Granuloma 2 | | | RIF, Granuloma 3 | | | RIF, Granuloma 4 | | |
| --- | --- | --- | --- | --- | --- | --- | --- | --- | --- | --- | --- |
| MSE= 0.02032 | | | MSE= 0.0096 | | | MSE= 0.0110 | | | MSE= 0.0076 | | |
| Exp. | Mod. | Diff. | Exp. | Mod. | Diff. | Exp. | Mod. | Diff. | Exp. | Mod. | Diff. |
| 1.0000 | 0.8196 | 0.1804 | 1.0000 | 0.8692 | 0.1308 | 1.0000 | 0.8447 | 0.1553 | 1.0000 | 0.8473 | 0.1527 |
| 0.8125 | 0.6095 | 0.2030 | 0.6468 | 0.6732 | -0.0264 | 0.8639 | 0.5935 | 0.2704 | 0.7457 | 0.5859 | 0.1598 |
| 0.6310 | 0.4430 | 0.1880 | 0.5153 | 0.5357 | -0.0204 | 0.6206 | 0.4348 | 0.1858 | 0.4914 | 0.4273 | 0.0641 |
| 0.4536 | 0.3314 | 0.1222 | 0.3766 | 0.4416 | -0.0649 | 0.4246 | 0.3308 | 0.0938 | 0.3103 | 0.3167 | -0.0064 |
| 0.3443 | 0.2585 | 0.0858 | 0.3323 | 0.3790 | -0.0467 | 0.2742 | 0.2543 | 0.0199 | 0.1746 | 0.2423 | -0.0677 |
| 0.2675 | 0.2069 | 0.0606 | 0.2757 | 0.3389 | -0.0633 | 0.1835 | 0.2058 | -0.0223 | 0.1078 | 0.1915 | -0.0837 |
| 0.2919 | 0.1701 | 0.1218 | 0.1751 | 0.3162 | -0.1411 | 0.1249 | 0.1702 | -0.0453 | 0.0754 | 0.1564 | -0.0810 |
| 0.2544 | 0.1453 | 0.1091 | 0.1408 | 0.3093 | -0.1685 | 0.1052 | 0.1437 | -0.0385 | 0.0496 | 0.1319 | -0.0823 |
| 0.2924 | 0.1273 | 0.1651 |  |  |  | 0.0845 | 0.1268 | -0.0423 | 0.0345 | 0.1140 | -0.0795 |
| 0.2779 | 0.1145 | 0.1634 |  |  |  | 0.0680 | 0.1138 | -0.0457 | 0.0216 | 0.1022 | -0.0806 |
| 0.2572 | 0.1056 | 0.1516 |  |  |  | 0.0515 | 0.1050 | -0.0535 | 0.0194 | 0.0938 | -0.0744 |
| 0.2460 | 0.0995 | 0.1465 |  |  |  | 0.0433 | 0.0993 | -0.0560 | 0.0172 | 0.0871 | -0.0698 |
| 0.2233 | 0.0954 | 0.1279 |  |  |  | 0.0392 | 0.0953 | -0.0562 | 0.0129 | 0.0826 | -0.0697 |
| 0.2118 | 0.0932 | 0.1186 |  |  |  | 0.0351 | 0.0931 | -0.0581 | 0.0129 | 0.0793 | -0.0664 |
| 0.2121 | 0.0924 | 0.1197 |  |  |  | 0.0268 | 0.0924 | -0.0656 | 0.0086 | 0.0771 | -0.0685 |
|  |  |  |  |  |  |  |  |  |  |  |  |
| CFZ, Granuloma 1 | | | CFZ, Granuloma 2 | | | CFZ, Granuloma 3 | | | CFZ, Granuloma 4 | | |
| MSE= 0.0135 | | | MSE= 0.0195 | | | MSE= 0.0037 | | | MSE= 0.0040 | | |
| Exp. | Mod. | Diff. | Exp. | Mod. | Diff. | Exp. | Mod. | Diff. | Exp. | Mod. | Diff. |
| 1.0000 | 0.8667 | 0.1333 | 1.0000 | 0.8730 | 0.1270 | 1.0000 | 0.8267 | 0.1733 | 1.0000 | 0.8529 | 0.1471 |
| 0.7407 | 0.6076 | 0.1331 | 0.8242 | 0.6715 | 0.1527 | 0.6432 | 0.5856 | 0.0576 | 0.6618 | 0.5924 | 0.0694 |
| 0.5279 | 0.4436 | 0.0844 | 0.6394 | 0.5356 | 0.1038 | 0.5276 | 0.4324 | 0.0953 | 0.3718 | 0.4271 | -0.0553 |
| 0.3566 | 0.3357 | 0.0209 | 0.4848 | 0.4436 | 0.0412 | 0.3392 | 0.3236 | 0.0156 | 0.2563 | 0.3171 | -0.0608 |
| 0.4284 | 0.2613 | 0.1671 | 0.3152 | 0.3801 | -0.0650 | 0.2286 | 0.2558 | -0.0272 | 0.2080 | 0.2410 | -0.0331 |
| 0.3648 | 0.2070 | 0.1578 | 0.3121 | 0.3395 | -0.0274 | 0.1734 | 0.2057 | -0.0323 | 0.1450 | 0.1921 | -0.0471 |
| 0.3510 | 0.1705 | 0.1805 | 0.3818 | 0.3167 | 0.0651 | 0.1759 | 0.1690 | 0.0068 | 0.0945 | 0.1569 | -0.0624 |
| 0.3206 | 0.1450 | 0.1756 | 0.0000 | 0.3093 | -0.3093 | 0.1683 | 0.1435 | 0.0248 | 0.0525 | 0.1323 | -0.0798 |
| 0.1797 | 0.1274 | 0.0522 |  |  |  | 0.0804 | 0.1256 | -0.0452 | 0.1113 | 0.1149 | -0.0036 |
| 0.2764 | 0.1146 | 0.1618 |  |  |  | 0.0754 | 0.1141 | -0.0387 | 0.0420 | 0.1028 | -0.0608 |
| 0.0525 | 0.1056 | -0.0531 |  |  |  | 0.0628 | 0.1051 | -0.0423 | 0.0734 | 0.0934 | -0.0200 |
| 0.1161 | 0.0995 | 0.0165 |  |  |  | 0.0829 | 0.0992 | -0.0163 | 0.0000 | 0.0868 | -0.0868 |
| 0.0719 | 0.0954 | -0.0235 |  |  |  | 0.0528 | 0.0954 | -0.0426 | 0.0462 | 0.0824 | -0.0361 |
| 0.0415 | 0.0932 | -0.0517 |  |  |  | 0.0452 | 0.0931 | -0.0479 | 0.0483 | 0.0791 | -0.0308 |
| 0.0001 | 0.0924 | -0.0923 |  |  |  | 0.0628 | 0.0924 | -0.0296 | 0.0546 | 0.0771 | -0.0224 |
