# Supplementary figures and images for: Mathematical model of oxygen, nutrient, and drug transport in tuberculosis granulomas
Source: PLoS Comput Biol. 2024 Feb 9;20(2):e1011847. doi: 10.1371/journal.pcbi.1011847 (PMC10883541; doi:10.1371/journal.pcbi.1011847)

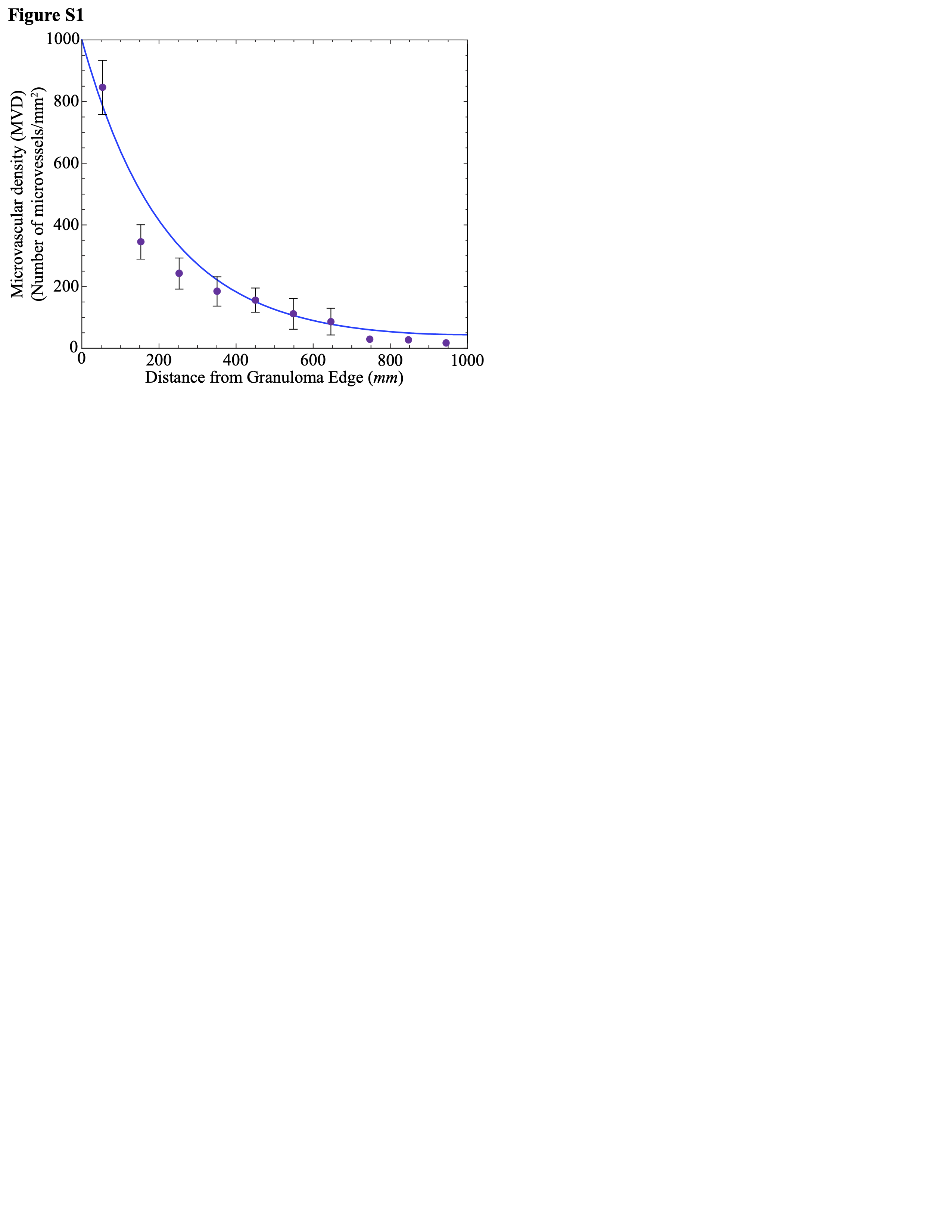

Supplement: S1 Fig — Comparison of experimental microvessel density (MVD; dots) in rabbit granulomas [5] versus fitting (lines) via Eq S34 (see S1 Text) for the following parameters: β = 1/2; R0 = 3 mm Lp = 2.8×10−7 cm∙mmHg−1∙s−1, Kv = 4.13×10^−8^ cm2∙mmHg−1∙s−1, Nv,0 = 1,000, and av,0 = 200 cm2∙cm−3. (TIFF) [file pcbi.1011847.s004.tiff]

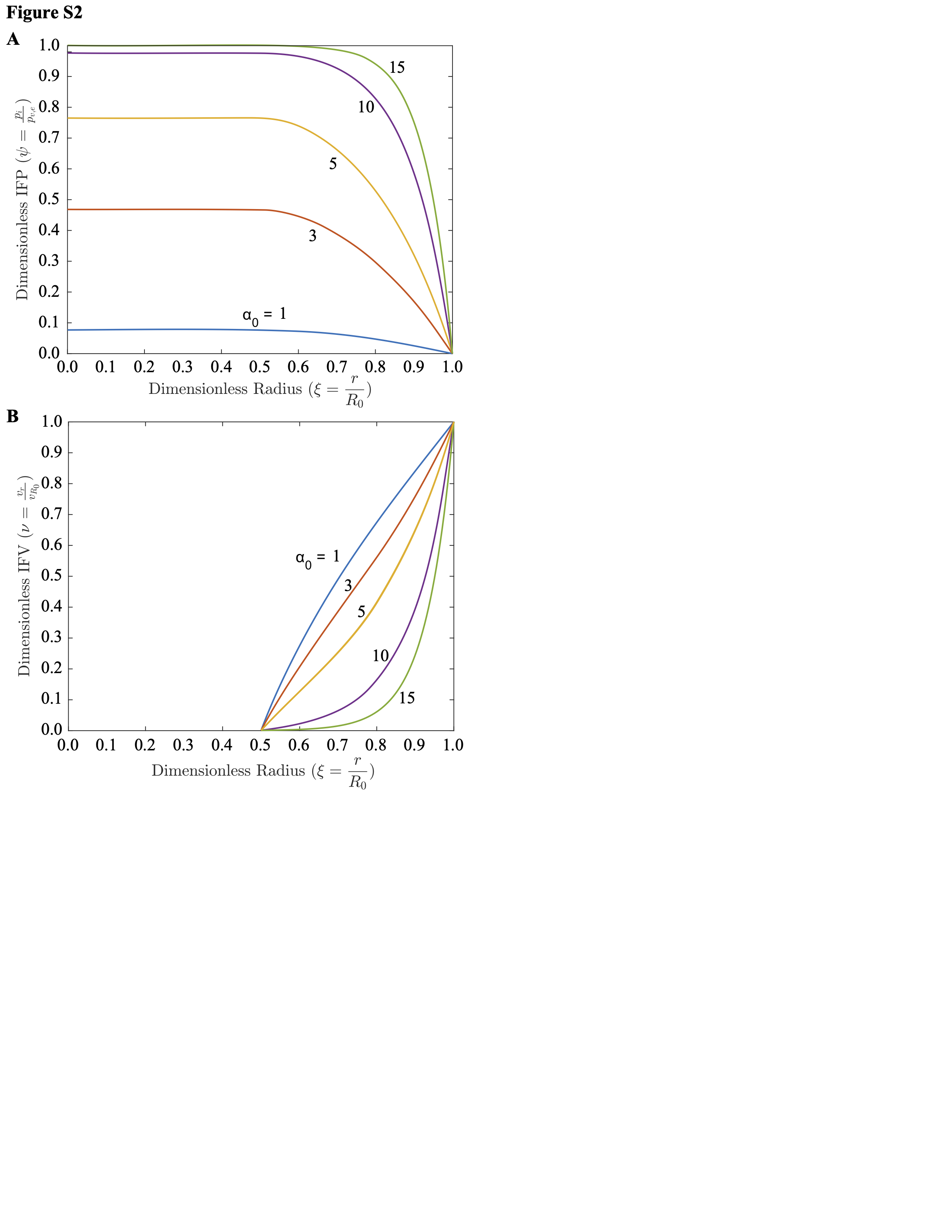

Supplement: S2 Fig — Predicted dimensionless (A) IFP and (B) IFP profiles within granulomas for different moduli α0 and for the shell-core perfusion model, with ξD = 0.5. (TIFF) [file pcbi.1011847.s005.tiff]

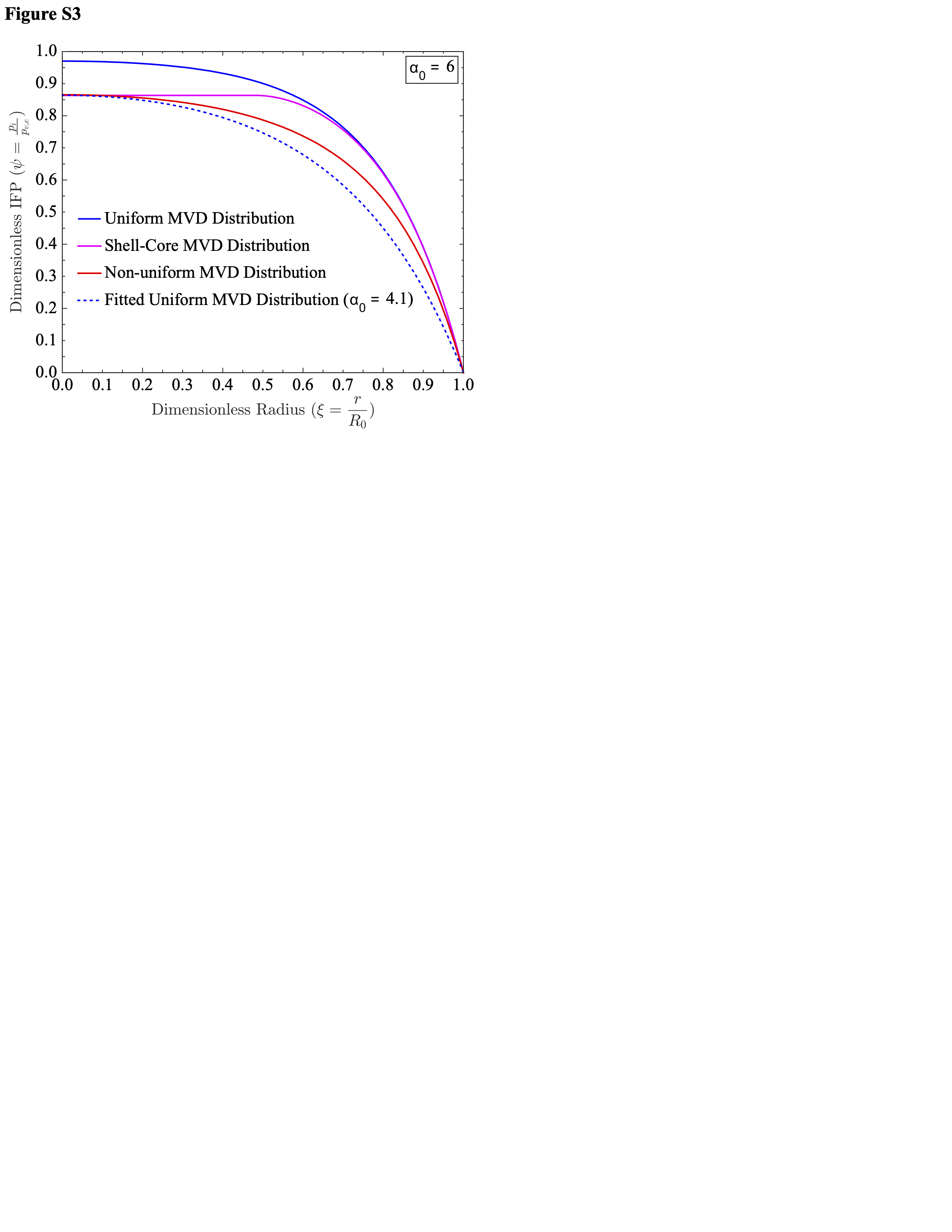

Supplement: S3 Fig — Predicted dimensionless IFP rise within granulomas for the uniform (Eq III), shell-core (Eq S25, see S1 Text), and non-uniform perfusion (Eq S35) models for case of α0 = 6, with an additional fitted uniform perfusion case for α0 = 4.1. (TIFF) [file pcbi.1011847.s006.tiff]
